# Slowly dispersing neotenic beetles can speciate on a penny coin and generate space-limited diversity in the tropical mountains

**DOI:** 10.1038/srep33579

**Published:** 2016-09-16

**Authors:** Timothy C. Bray, Ladislav Bocak

**Affiliations:** 1 Department of Zoology, Faculty of Science, Palacky University, 17. listopadu 50, 771 46 Olomouc, Czech Republic; 2 Institute of Conservation Science and Learning, Bristol Zoological Gardens, Bristol, Clifton BS8 3HA, UK

## Abstract

We demonstrate the controversial origin of a biological species within an area of a few kilometres in the absence of physical barriers. We employed nuclear rRNA/mitochondrial and genome-wide SNP approaches to infer relationships of four species of net-winged beetles characterised by female neoteny. Three species are distributed at low elevations and a single population colonised a 40 km^2^ highland plateau and established distinct biological species despite incomplete genetic isolation. The speciation process is extreme in the highly localised spatial scale, due to the low dispersal power of neotenics, and provides clear support for a microallopatric model based on ecological conditions. In contrast with neutral evolution in a homogenous environment, as demonstrated by the genetic divergence and morphological similarity of two widely distributed low-mountain species, the environmental characteristics of the high-mountain plateau led to the origin of a species adapted to the local mimetic pattern and characterised by morphologically distinct genitalia. We conclude that the low dispersal propensity promotes neutral genetic differentiation in the first stage, but environmental characteristics play an important role the final phase of the speciation process. The unexpected speciation at such an extreme geographic scale points to the *in situ* origin and uniqueness of the mountain fauna.

A large part of insect diversity remains unrecognised especially when genetic diversity is decoupled from morphological differentiation^1^,^2^,^3^ and when species occur in very small ranges and in under-investigated regions^4^. Studies of speciation processes limited to an extremely small spatial scale are needed to understand the extent and spatial distribution of diversity, species delimitation, and to identify the processes which produce endemism in limited areas, e.g., in the tropical mountains^5^,^6^.

The common speciation model suggests allopatry over a large geographic scale and with prominent isolation barriers preventing gene flow^7^. Alternatively, various animal lineages with limited dispersal power readily diversify within much smaller areas without clear geographic or ecological barriers with neutral evolution being an important component generating their geographically structured genetic diversity^8^,^9^. The neotenic lineages are an extreme example of animals with very low dispersal propensity as they readily diversify within limited areas and so can be used as a model system to demonstrate the effect of neoteny and flightlessness on the speciation process^8^. Besides neutral processes, additional factors can support differentiation such as habitat diversity^10^ or temporal, spatial^11^, and ecological separation^12^ in a single ecosystem. Nevertheless, these factors cannot be decoupled from dispersal propensity which determines the intensity of gene flow between populations and consequently at what scale the deep genetic divergence leading to speciation can occur. Speciation across a small geographical range has been considered as an uncommon scenario^7^, but detailed studies can challenge this expectation. We propose that extremely low dispersal propensity can be the principal factor rescaling the meaning of the geographical distance and might be a primary cause for the observed high speciation rate in poor dispersers^8^.

The divergence caused solely by neutral processes is slow and only a stable and continuous environment can provide sufficient space and time for high levels of genetic differentiation. The optimal areas for studies dealing with poor dispersers are tropical regions where the communities represent extremely diversified assemblages with a long evolutionary history^13^. Here, we study the fauna of the Malay Peninsula, one of the most stable tropical regions with regard to tectonic history and position close to the equator^14^,^15^. The tropical rainforests depend on climatic conditions which are generally unstable^16^, but the monsoon dependant rainforests in South East Asia have long history starting with the origin of the monsoon system after the collision of India and Asia^17^,^18^. Although the extent of tropical rainforests dynamically changed due to climate fluctuations since their origin^19^, the high mountains along the western margin of the Sundaland obtained higher rainfall than central lowlands of the subaerial Sunda Shelf even during periods of extreme aridification^20^.

We need to expand the spectrum of model groups to investigate the power of individual factors in the speciation process, but research using non-model organisms has suffered from a restricted development of appropriate molecular marker systems with the resolution to study the origins of reproductive isolation. Therefore, the net-winged beetle neotenics have been omitted, although they are a promising model for differentiation and speciation studies. All of them have uniform ecology and occur in ecologically stable habitats which enable long-term survival of animals with low dispersal propensity^21^,^22^ and they are known for their very low dispersal propensity and low flying activity of males due to presence of larviform females^23^. Here, we present a clade of four closely related species of *Scarelus* net-winged beetles (Fig. 1) as a model system for diversification at an extremely small geographic scale. A series of studies has been recently published on neotenic beetles including *Scarelus* and most of them focused on diversification in large areas and over long time spans^23^,^24^. The phylogenetic analyses identified the deeply split clades of species occurring in each region, the geographically structured species distribution even within individual areas^23^,^24^, and in all cases the ranges of neotenics are limited to long-term stable ecosystems^22^. Although not as brightly coloured as other unpalatable^25^ net-winged beetles, *Scarelus* colouration regularly fits in the local mimetic complexes of under-canopy dwelling net-winged beetles and their mimics. Colour patterns adapted to syntopically occurring mimetic complexes have been hypothesised as factors starting the speciation process in net-winged beetles previously^26^, with the aposematic signal also shown to constitute a barrier to gene-flow in other animal groups^27^. Conversely, the adult morphology of *Scarelus* is uniform and differences are limited to the shape of male genitalia and length of antennae^28^.

Here, we describe an extremely spatially limited diversification process within the Main Range of the Malay Peninsula, where no clear barriers are present on a continuous slope of the Cameron Highlands massif. We re-visit a previously identified *Scarelus* system characterised by female neoteny^23^ and study in detail a clade of closely related species: a single species is of black colouration with an ‘island’ distribution on a highland plateau surrounded by varying degrees of light coloured species at lower elevations. We use large numbers of anonymous genetic markers (RAD) as well as mitochondrial and nuclear markers to ask whether it is possible to identify genetic structure within this system, including the origin of the highland species and to see if adaptation to local mimicry patterns and morphology fits with genetic divergence and plays a role in the diversification process.

## Results

### Sanger and NextRAD sequencing, filtering and SNP calling

Three mtDNA fragments, *rrnL–tRNA-Leu–nad1, cox1–tRNA-Leu–cox2*, and *nad5–tRNA-Phe–tRNA-Glu–tRNA-Ser*, were obtained for 42 specimens from the Main Range and combined with earlier available nuclear rRNA and mitochondrial DNA data^22^,^23^. The aligned dataset consisted of 18S rRNA (1865 homologous positions), 28S rRNA (643 positions), *rrnL* mtDNA (793 positions), *cox1* mtDNA (1109 positions), and *nad5* mtDNA (1187 positions). We identified up to 17 unique haplotypes in individual mtDNA fragments for a single species (Supplementary Table S4) and the uncorrected mtDNA pairwise distances among *Scarelus* species reached up to 21.55% (Supplementary Tables S3, S5–S7).

The nuclear NextRAD data comprised 88–147,000 clusters of potential loci across individuals with a mean depth 9–22 reads per cluster. Raising the clustering threshold increased the number of clusters recovered and causing within-individual heterozygosity to drop sharply above the value 0.8 (Supplementary Figure S5). This trade-off between heterozygosity and cluster number was used to choose a threshold of 0.8. This value is slightly higher than the heterozygosity peak as the introduced bias from erroneous cluster-splitting has been deemed relatively unimportant compared with that from over-clustering, and potentially allows the recovery of more loci. A similar trade-off between numbers of accepted clusters and the stabilisation of heterozygosity was used to infer a clustering depth of 8 for the population genetic analyses (Supplementary Figure S4). Final data analysis was performed using the parameters mindepth = 8 clustering threshold = 0.8, minimum 21 individuals to accept locus. For the final dataset of 25 individuals the read numbers and mean coverage per individual were calculated (Supplementary Table S8). Raw reads for all individuals were deposited in GenBank under SRA accession number SRR3679806. We allowed up to 25% missing data to obtain the higher numbers of loci and SNPs in the dataset. The resultant nuclear datasets for the final analyses gave between 92 and 1853 loci comprising 593–12737 SNPs. The SNP data showed 72.90–87.75% identical bases in the 3-species dataset (Supplementary Table S9). From 1354 independent rad tags (loci) tested for signal of selection across all individuals, BAYESCAN determined that no loci displayed signal of selection. The LOSITAN approach suggested that across all individuals 11 loci were under positive selection with 27 loci showing signal of balancing selection. The samples of distantly related *S. umbrosus* were removed and the tests were repeated across 1777 loci for the remaining three species. The signal of positive selection was seen across 24 loci and balancing selection in five and all of these loci were excluded from subsequent analyses.

### Species delimitation

**Figure 2:**
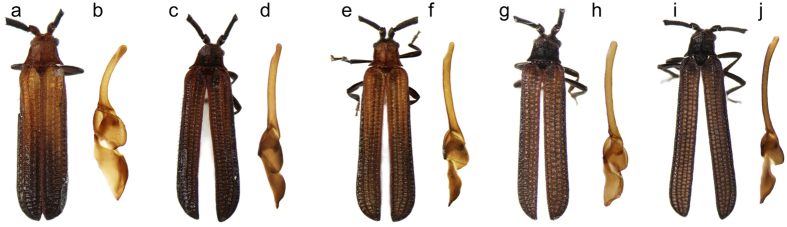
General appearance and male genitalia of *Scarelus*. (**a,b**) *S. umbrosus*, (**c,d**) *S. pahangensis*, (**e,f**) *S. pseudoumbrosus*, (**g,h**) *Scarelus* sp., individual NG0038, (**i,j**) *S. anthracinus*. (see Supplementary Figure S9 for further information).

**Figure 3:**
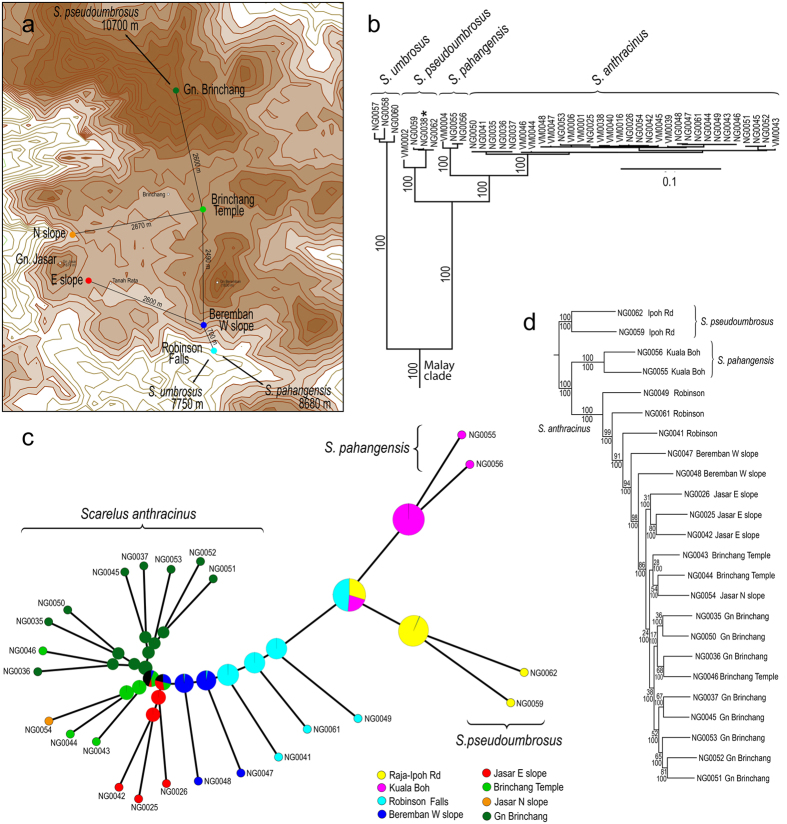
(**a**) Map of the Cameron Highlands Plateau and adjacent peaks, the distances given for localities sharing related populations of *Scarelus anthracinus*. The map was produced using the QGIS software package (www.qgis.org/en/site/forusers/download.html) from ASTER GDEM V2 data publicly available from the Land Processes Distributed Active Archive Center (LP DAAC) and J-spacesystems. (**b**) Phylogenetic hypothesis for the Malay clade of *Scarelus* based on a maximum likelihood (ML) analysis of five fragments produced by Sanger sequencing. (**c**) The Bayesian reconstruction of ancestral areas for populations of three species from the Cameron Highlands massif inferred from the RAD dataset. (**d**) Phylogenetic relationships for populations of three *Scarelus* species from the Cameron Highlands massif based on the maximum likelihood analysis of the 24-sample RAD dataset. The sample NG0038 omitted, see Supplementary Figure S7 for comparison.

Four closely related species were identified in the Cameron Highland massif and their limits are supported by nuclear and mtDNA datasets. High intraspecific genetic variability was identified in all species and reached up to 0.76, 3.17 and 2.61% for *rrnL, cox1* and *nad5*, respectively (Supplementary Table S4). The interspecific pairwise *cox1* distances between *S. anthracinus* and *S. pahangensis*/S*. pseudoumbrosus*, reached 5.74–6.53% and 8.93–10.03%, respectively (Supplementary Table S6, Figure S3). Additionally, the species limits are supported by differences in male genitalia (Fig. 2, Supplementary Figure S9) and colouration of elytra and pronotum which subtly varies within the lowland brown pattern (Fig. 2); only *S. anthracinus* (Fig. 2i) belongs to a black colour pattern which is endemic to high elevations in the Cameron Highlands. The identified hybrid specimen NG0038 shared mtDNA sequences with *S. pseudoumbrosus* (Fig. 3b), but its nuclear DNA pointed to much closer relationships with *S. anthracinus* (Supplementary Figure S7a). The specimen has intermediate morphology and cannot be formally classified with either of the parental species (Fig. 3a, Supplementary Figure S7a,b, Supplementary Table S9).

**Figure 4:**
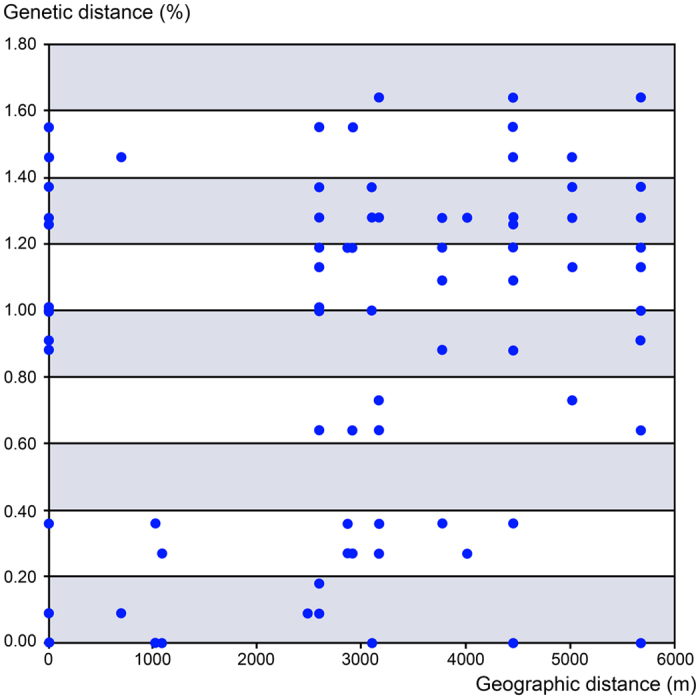
Intraspecific relationships between uncorrected pairwise genetic and Euclidean distances of *Scarelus anthracinus*.

The STRUCTURE analysis of the dataset of 24 individuals after removing *S. umbrosus* cluster resulted in 1746 SNPs. At K = 3 the three groups separated with high memberships to their own clusters (Q_mean_ > 0.80), i.e. *S. pseudoumbrosus, S. pahangenis*, and *S. anthracinus*. This mirrors the species partitioning seen in the PCA (Supplementary Figure S6B). Two species, *S. pseudoumbrosus* and *S. pahangensis* were seen to be less differentiated relative to each other (F_ST_(W&C 1984) = 0.17) than either compared with *S. anthracinus* (F_ST_(W&C 1984) = 0.28 for both). Using the POPFLAG admixture determination for the putative hybrid individual NG0038 gave a majority contribution from *S. anthracinus* with smaller contributions from the other two species (~0.78, 0.14, 0.08, respectively). The RAD genetic distances among of 19 individuals of *S. anthracinus* of were moderately positively correlated with geographical distances (correlation coefficient *r* = 0.528, *P* ≤ 0.001, Fig. 4).

### Sanger and SNP phylogeny, dating and morphology

The maximum likelihood (ML) analysis of the dataset mitochondrial DNA and nuclear rRNA fragments produced the tree with *S. umbrosus* as a sister to the clade (*S. pseudoumbrosus (S. pahangensis, S. anthracinus*; Fig. 3b). The partial Bayesian analyses of *cox1* and *rrnL* fragments recovered a similar topology and inferred the split of *S. pseudoumbrosus* 5.39 and 3.47 mya, *P. pahangensis* versus *S. anthracinus* 2.17 and 1.92 mya and the most basal split among *S. anthracinus* populations 0.409 and 0.681 mya, respectively; Figure S8a,b).

The nuclear ML phylogeny inferred from the SNP dataset was similar to mtDNA/rRNA phylogeny (Fig. 3c,d, Supplementary Figure S7a,b), except the position of the individual NG0038 within *S. anthracinus* clade in contrast with its position within *S. pseudoumbrosus* clade in mtDNA phylogeny (Fig. 3b). The BS support for deep splits in the SNP phylogeny was slightly lower when the putative hybrid NG0038 was included in the analysis (Supplementary Figure S7a,b). The same topology was recovered in the Bayesian analyses of the same dataset with high posterior probabilities for all recovered clades (Fig. 3d).

The area of supposed split between *S. pahangensis* and *S. anthracinus* is located on the southern slope of Gunung Beremban within an area of a few square kilometres between Robinson Falls and Kg. Kuala Boh (Supplementary Figure S1a,b). The genetic diversity in mtDNA markers suggests intraspecific differentiation during a few hundred years (0.4–0.7 mya, Supplementary Figure S8). The “footprint” of the dispersal directions within the *S. anthracinus* clade was identified only in the nuclear data when the first splits among *S. anthracinus* populations were identified along on the southern slope of Mt. Beremban in very close proximity to the range of its sister-species *S. pahangensis* (8.68 km away, Fig. 3a,c) and shallower splits were inferred gradually on the western and northern slope of Mt Beremban and further populations reached Mt. Brinchang in the north and Mt. Jasar in the west. The most distant *S. anthracinus* populations were collected 5.6 km apart (Fig. 3a, Supplementary Table S3).

## Discussion

The lycid neotenics are known for extremely low dispersal propensity and complete species turnover between individual mountain ranges^23^,^24^. Here, for the first time we study their diversification process at an extremely fine geographic scale of a few kilometres in the Cameron Highlands Plateau and adjacent peaks. We assume that mountain populations of *S. anthracinus* became genetically separated from the nearby occurring lowland species by both distance and environmental differences. As these beetles develop in soil and females remain larviform, the barriers ineffective in restricting other flying insects such as small mountain streams, small breaks in forest cover, rock massifs or forest heterogeneity due to microclimatic conditions can substantially limit their dispersal. Given the distances <15 km between localities of all four allopatrically distributed species in the Cameroon Highlands massif (Fig. 3, Supplementary Table S3, Supplementary Figure S1), we consider it to be very unlikely that they have ever been completely geographically isolated by anything other than such non-permanent and small-scale kinds of barriers. Therefore we conclude that the speciation process proceeded without geographic isolation. The predicted ability to form genetically divergent subpopulations at small geographic scales is supported by the observed high genetic intraspecific divergence in *S. anthracinus* (Supplementary Table S4–S7, Figure S3). The role of distance in diversification is well known^29^, but the *Scarelus* system substantially differs in the extremely small scale at which this differentiation proceeds (Supplementary Table S3).

Our data are consistent with the single dispersal event leading to separation of *S. anthracinus* from *S. pahangensis* occurring in the lower elevations on the eastern slope of the Main Range (Fig. 3). We expect the primary hybrid zone between the middle part of the valley north of Sungai Bertam (locality of *S. pahangensis*) and the south-eastern margin of the Cameron Highlands plateau (Robinson Falls, 8.7 km away, Supplementary Figure S1). The SNP phylogeny identifies the individuals from the Robinson Falls among the deepest splits within the *S. anthracinus* clade and the dispersal directions can be identified to the north and east with distance steps <3 km (Fig. 3c). Our identification of the place, where the first splits among the populations of *S. anthracinus* are inferred, is supported by the adjacent range of the putative sister species, *S. pahangensis*. The dated phylogeny (Supplementary Figure S8) suggests very slow dispersal across a few kilometres of the plateau (0.41 mya since the basal split in the *S. anthracinus* clade; Fig. 3a–d, Supplementary Figure S8). Even if a substantially higher *cox1* rate might be considered for poor dispersers^21^,^30^, the time needed for the colonisation of the Cameron Highlands plateau would be estimated in tens of thousand years. The 5.6 km distance between the localities of the inferred deepest split of *S. anthracinus* populations and the terminal split in the Gunung Brinchang area (Fig. 3c) means that the average dispersal rate is extremely low and can be estimated in centimetres per year. Such dispersal rate is at least ten times lower than previously reported rate for such poor dispersers as minute land snails^31^.

The split between *S. anthracinus* and two closely related species is recent (Supplementary Figure S8) and not surprisingly, ongoing gene flow was identified among *S. anthracinus* and *S. pseudoumbrosus* when the putative hybrid specimen with intermediate morphology was collected in the contact zone between respective ranges (Fig. 3a, Supplementary Figure S1). Nevertheless, we consider such cases of introgression as rare and not threatening the morphological and genetical coherence of the *S. anthracinus* as we collected further 78 individuals and using morphology, all these samples were easily categorised as *S. anthracinus* using colouration and male genitalia (Fig. 2i,j).

Besides the process of neutral divergence^3^,^9^, two further factors could start genetic differentiation and subsequently keep coherence of the incipient species: (1) almost all highland net-winged beetles and their mimics are black and this colour pattern was adopted by *S. anthracinus* (Fig. 2i). The net-winged beetles are protected by the presence of pungent and slightly poisonous substances^32^ and they form local mimetic complexes. The endemic colour pattern can limit survival outside the range^33^ and act as an isolating mechanism^26^. Low intraspecific variability of colouration suggests the presence of selection for similarity within mimetic complexes. (2) The seasonal activity of the adult net-winged beetles strongly depends on moisture due to limited sclerotisation. The mountain plateau has similar rainfall to the lower elevations (Supplementary Figure S2c) but the high level of humidity is also maintained by water trapped by condensation in the cloud covered plateau and due to the lower temperatures (Supplementary Figure S2b). Consequently, organic material, in which all *Scarelus* develop, heavily accumulates on the soil surface and *Scarelus* is much more common in higher elevations and active throughout the year. Unlike this favourable habitat, *Scarelus* are rare and seasonal in the lower elevations (<1000 m, field observations, Supplementary Table S1). *S. pahangensis* occur on the eastern slope of the Main Range, where the autumn monsoon is stronger and the rest of the year has long dry periods during which their activity is limited or absent (Supplementary Figure S2d). This additional factor might limit opportunity of the highland species to encounter a mate from the parental population despite their geographical proximity.

We observe an extremely narrow zone where a subpopulation splits off from the parental species and established a biological species when ecological factors were the only variable parameters and they changed gradually with elevation. The low dispersal propensity and resulting low gene flow are characteristic for all four species. We assume that low dispersal power alone lead to genetic differentiation without morphological and ecological divergence as seen e.g. in high genetic divergence within *S. umbrosus* and the lower genetic differentiation between *P. pseudoumbrosus* and *P. pahangensis* than between either of them and *S. anthracinus.* Similar geographically structured differentiation and morphological uniformity as among lowland *Scarelus* was identified in other net-winged beetles^3^. Therefore, we conclude that ecology is the necessary component supporting morphological differentiation and origin of the new species in the *Scarelus* system. Low gene flow in the neotenic lineages defines the extremely small geographic scale, but alone it leads to gradual, neutral evolution and such process does not necessarily lead to the clear morphological differentiation and the origin of a biological species^3^.

The net-winged beetle neotenics occur exclusively in the stable habitats^22^ and have regularly small ranges^23^,^24^. The observed pattern can be a result of frequent loss of their diversity in unstable habitats due to deep disturbances such as extensive desertification. The elateroid lineages contain numerous neotenic lineages and their independent, relatively frequent origin is well supported^34^,^35^,^36^. Nevertheless, their current diversity is limited to the humid tropics^22^ or they become soil-dependent if they survive in the less humid but otherwise stable ecosystems such as the Mediterranean (e.g. Omalisidae and Iberobaeniidae^34^,^37^). As an alternative to the selection by habitat conditions, we propose that they evolve in any temporarily favourable ecosystems, but survive for long only in those with long stability. Only then we can observe the deep diversification within the clades of the non-flying beetles^23^,^24^. Therefore, the stable habitats can be museums of diversity predominantly due to habitat stability leading to the lower extinction rate^38^.

The winged insects can colonise distant regions^39^, they easily diversify by multiple rapid altitudinal shifts^5^,^6^ and simultaneously they can effectively react if new dispersal opportunities are available^40^. The *Scarelus* lineage with their forest dependent immobile neotenic females represents a different and very unusual system. Their ecological characteristics permits only very slow dispersal accompanied by continuous microallopatric differentiation within very small regions (Figs 2 and 4). As a result the ranges of newly split species can be as small as a few dozens of km^2^ and the likelihood of extinction in the changing environment is much higher than in mobile animals keeping dispersal power. Therefore, the occurrence of diversified neotenics in an area can serve as an indicator of uninterrupted evolutionary history of highly productive tropical ecosystems. The processes of the space-limited differentiation lead to the observed high level of endemism in the Cameron Highlands mountain “island” which can be easily destroyed either by human activity or climate fluctuation.

## Methods

### Sampling and Sanger sequencing

Four species of *Scarelus* net-winged beetles (Coleoptera: Lycidae: Ateliinae) from the Cameron highlands in the Malay Peninsula were included in the combined mtDNA and rRNA phylogeny of the whole genus: *Scarelus anthracinus* (31 spec.), *S. pahangensis* (3 spec.), *S. pseudoumbrosus* (4 spec.), and *S. umbrosus* (3 spec.). All four species were collected in an area of ~400 km^2^ in elevations 500–1900 m above sea level and only a single species was recorded in each locality (Table 1, Fig. 3, Supplementary Table S1, Supplementary Figure S1). DNA was extracted from the metathoracic muscles of each specimen using a DNeasy tissue kit (Qiagen). Three mitochondrial fragments were amplified: *rrnL–tRNA-Leu–nad1* (~760 bp), the 3′end of *cox1–tRNA-Leu–cox2* (1090 bp), and *nad5–tRNA-Phe–tRNA-Glu–tRNA-Ser* (~1150 bp; for primers and conditions see Supplementary Table S2). The fragments are further referred as *rrnL, cox1*, and *nad5*, only. The PCR products were purified using PCRu96 Plates (Millipore) and sequenced by an ABI 3130 automated sequencer using the Big Dye Sequencing Kit 1.1 (Life Technologies). The chromatograms produced by Sanger sequencing were edited using Sequencher 4.8 (Genecodes Inc.) and the new data (GenBank accession codes KU319589–KU319671) were merged with the previously published dataset containing additionally full-length18S rRNA (~1855 bp) and the D2 region of 28 S rRNA (~630 bp) for all species included in the analysis^23^,^24^.

### NextRAD sequencing, filtering and SNP calling

High numbers of single nucleotide polymorphisms (SNPs) were used to investigate relationships among closely related species of *Scarelus* and their populations. Such an approach has the power to detect even low level consequences of demography and introgression^41^, enables a study to use a non-model group^42^ and provides sufficient data to make such analyses feasible^43^,^44^. The set of 28 samples representing individuals of four species (*S. umbrosus, S. pseudoumbrosus, S. pahangensis*, and *S. anthracinus*) and their populations from 11 localities in the Cameron Highlands was prepared for RAD sequencing (Table 1, Fig. 3a, Supplementary Figure S1). The samples were processed for nuclear markers with the NextRAD genomic sequencing approach (SNPsaurus) using the Illumina HiSeq 2500 system. NextRAD sequencing produces primer-based individually barcoded single-end read amplifications with average length ~75 bp representing loci scattered across the genome. These data were processed for all individuals with separate identifiers. The pyRAD 3.0.4^45^ workflow was employed to perform the filtering and SNP recovery process. Data were evaluated in order to maximise the numbers of SNPs recovered and in so doing minimise any bias introduced by the filtering process^46^,^47^. Initial full runs (all individuals) were used to select a reduced representation of six individuals representing the spatial extent of the sampling which were then run at a range of clustering thresholds between 65 and 90%. Within individual heterozygosity scores were averaged and these were used to evaluate clustering success^48^. A sensitivity analysis was subsequently conducted to ascertain the influence of the minimum coverage depth required for locus inclusion (values of 4–9). Because of the large divergence of *S. umbrosus* individuals, a 25-sample dataset was created excluding three *S. umbrosus* samples. Analysis of this subset of data allowed a higher proportion of accepted marker loci for further analyses. Some processing and calculations for the methods were run through R^49^. Required file formats not generated in pyRAD were converted by PGDSpider^50^.

We tested all loci for signal of selection across populations in order to remove those potentially violating the assumption of neutrality. We applied two F_ST_ outlier approaches performed in BAYESCAN^51^ and LOSITAN^52^. The methods use the divergence in allele frequencies between populations and a common gene pool to generate a subpopulation specific F_ST_ coefficient. These analyses were performed on four- and three-species datasets, with and without *S. umbrosus*.

We further employed a population genetic approach to investigate introgression using the STRUCTURE^53^ application and the Principal Component Analysis (PCA)^54^. Genetic cluster proportions over populations were averaged across 20 runs at 10^5^ steps of burn-in followed by 10^5^ steps for data collection. The individual NG0038 was identified by PCA centrally positioned with no clear group membership in the 25-sample dataset and an estimate of admixture proportions was calculated for this individual against the three genetic clusters representing *S. pseudoumbrosus, S. pahangensis*, and *S. anthracinus*. Population genetic summary statistics were calculated in each species for the NextRAD data in R and population differentiation information measured in 4P^55^. Two datasets were considered; all individuals of four species and a dataset excluding *S. umbrosus*. Further, data matrices were assembled to compare geographical distances and RAD genetic distances among 19 individuals of *S. anthracinus*. The correlation coefficients for geographic and RAD genetic distances and their corresponding P-values (Mantel tests, number of repetitions nrepet = 10000) were analysed in R 3.1.1^49^.

### Phylogenetic analyses of Sanger data and mtDNA based dating

Nuclear rRNA and mitochondrial DNA fragments were aligned using default parameters of Mafft 7.2^56^ and the alignments of the protein coding genes were checked for amino acid reading frames. We conducted Maximum Likelihood (ML) phylogenetic analyses using RAxML-HPC2 8.1.24 as implemented in the CIPRES web server^57^,^58^ with data partitioned by genes and by codons, respectively (18 partitions). The analysis was performed using 100 searches for the best tree under a GTR + G + I model identified using jModelTest 2^59^, with bootstrap (BS) values calculated using the rapid algorithm^60^ with 1,000 bootstrap iterations under the GTRCAT model. We dated the splits between species using single-fragment datasets for the Cameron Highlands *Scarelus* clade in Beast 1.8.1^61^ using the GTR + G + I model identified as above, Yule Process, and Lognormal Uncorrelated Relaxed Clock as proposed in the Beast manual^62^. The molecular clock was tested for *cox1* and *rrnL* mtDNA datasets using a likelihood ratio test. The null hypothesis L_0_ is that molecular clock holds and L_1_ hypothesis relaxes the clock constraint. The chi-square value is given by 2logL = 2(logL_0_ − logL_1_) where L_0_ and L_1_ are likelihoods of the tree under given constraints. The p-value is counted for s-2 degrees of freedom where s is the number of terminal branches on the tree^63^. The null model was rejected and an Uncorrelated Relaxed Clock was used in the analyses. As the fossils are unavailable, the 0.0177 ± 0.0019 substitutions/site/my/lineage for *cox1* fragment and the 0.0054 ± 0.0009 subs/s/my/l for *rrnL* fragment^21^ were used for calibration. The MCMC parameters were fixed to 30 million generations with sampling every 10,000 generations and Effective Sample Size values (ESS) and the pre-stationary phase were identified in Tracer 1.6^64^. The initial generations were discarded as burn-in.

### SNP based phylogeny and reconstruction of ancestral distribution

The datasets of SNP sequences representing three Cameron Highlands species and 25 or 24 individuals were produced for phylogenetic analyses (Table 1). *S. umbrosus* was excluded from both datasets due to the high level of missing data and alternatively the sample NG0038 due to the putative hybrid origin. The NextRAD data were used for all individuals, accepting up to 25% missing data. Datasets were analysed under the ML criterion using RAxML-HPC2 8.1.24 as implemented in the CIPRES web server^57^,^58^ and data were unpartitioned. The analyses were performed using 100 searches for the best tree under a GTR + G model identified using jModelTest 2^59^, with bootstrap (BS) values calculated using the rapid algorithm^60^ with 1,000 bootstrap iterations under the GTRCAT model. Additionally, we used the Bayesian inference implemented in the Beast 1.8.1^61^ to construct the phylogenetic tree from the 24-sample dataset using the HKY model^62^, Birth-Death process, Strict and Lognormal relaxed clock^50^, respectively, and the clock rate fixed to 1.0. The analyses using the GTR + G model proposed by jModelTest 2^59^ did not reach a stationary phase, therefore the second best identified model was applied in the Bayesian analyses. The stationary phase and ESS values were evaluated in Tracer 1.6^64^. Additionally, the ancestral areas were inferred in Beast 1.8.1^61^ using the HKY model as above, Coalescent Model for the speciation process, Constant Population Size, Strict Clock and constrained topology gained from the ML analysis of the same dataset. The parameters of the analysis follow the recommendation in the Beast manual^62^. The localities were coded as given at terminals in Fig. 3c.

### Morphology

The morphological characteristics of each sampled individual were investigated using external morphology and male genitalia. The dry preserved collection and sequenced individuals contained *S. anthracinus* (78 spec., all from the Cameron Highlands), *S. umbrosus* (6 spec. from the Fraser Hills, Gn. Benom, and Tapah–Ringlet Rd), *S. pseudoumbrosus* (6 spec. from Korbu Mts and Ipoh–Kg. Raja Rd) and *S. pahangensis* (5 spec. Kg. Kuala Boh and Mt. Benom). Genitalia were dissected, cleaned from muscles and fat bodies and photographed using a camera attached to a binocular microscope.

## Additional Information

**How to cite this article**: Bray, T. C. and Bocak, L. Slowly dispersing neotenic beetles can speciate on a penny coin and generate space-limited diversity in the tropical mountains. *Sci. Rep.* **6**, 33579; doi: 10.1038/srep33579 (2016).

## Supplementary Material

Supplementary Information

## Figures and Tables

**Figure 1:**
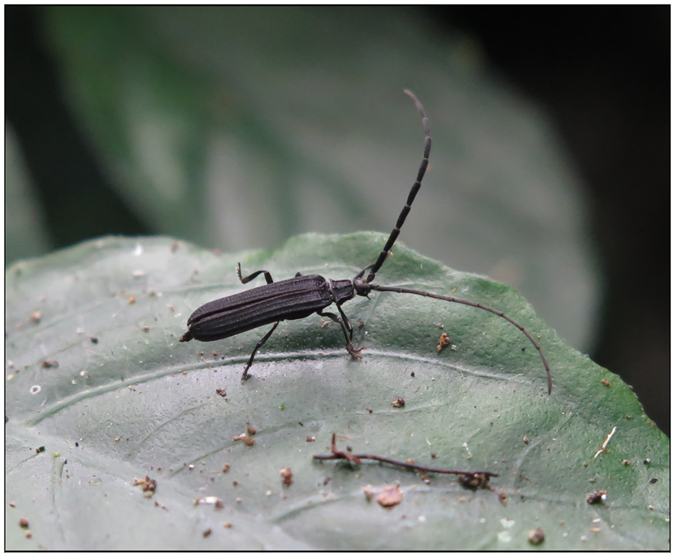
*Scarelus anthracinus* (Coleoptera: Lycidae) in nature.

**Table 1:** The list of *Scarelus* samples included in the RAD analysis (Locality names in parentheses are used in Fig. 3).

Species	Voucher Number Locality data (all Malay Peninsula)	
*S. anthracinus*	NG0041, 49, 61	Pahang, Gn. Beremban, 1270 m, 4°27′ 31″N 101°23′35″E (Robinson Falls)
	NG0047–48	Pahang, Gn. Beremban, 1480 m, 4°27′ 51″N 101°23′26″E (Beremban W slope)
	NG0025–26, 42	Pahang, Gn. Jasar, E slope, 1500 m, 4°28′23″N 101°22′06″E (Jasar, E slope)
	NG0043–46	Pahang, Gn. Beremban, N slope, 1580 m, 4°29′ 12″N 101°23′27″E (Brinchang Temple)
	NG0035–37, 50–54	Pahang, Gn. Brinchang, 1800 m, 4°30′ 34″N 101°23′09″E (Gn. Brinchang)
*Scarelus* sp.	NG0038	Pahang, Gn. Jasar N slope, 1550 m, 4°28′ 56″N 101°21′55″E (Jasar, N slope)
*S. pahangensis*	NG0055–56	Pahang, Kampong Kuala Boh, 960 m, 4°26′18″N 101°28′07″E (Kg. Kuala Boh)
*S. pseudoumbrosus*	NG0059, 62	Perak, km 47, Rd. Ipoh–Kg. Raja, 1100 m, 4°35′ 32″N 101°18′50″E (Ipoh Rd)
*S. umbrosus*	NG0060	Perak, km 24, Rd Tapah–Ringlet, 557 m, 4°19′29″N 101°19′21E
	NG0058	Perak, km 34, Rd Tapah–Ringlet, 610 m, 4°22′16″N 101°20′0″E
	NG0057	Perak, km 40, Rd Tapah–Ringlet, 1030 m, 4°23′30″N 101°22′19″E
