# Supplementary material for: Slowly dispersing neotenic beetles can speciate on a penny coin and generate space-limited diversity in the tropical mountains
Source: Sci Rep. 2016 Sep 16;6:33579. doi: 10.1038/srep33579 (PMC5025657; doi:10.1038/srep33579)
Supplement: Supplementary Information [file srep33579-s1.pdf]

Slowly dispersing neotenic beetles can speciate on a penny coin and generate space-limited diversity in the tropical mountains

Timothy C. Bray and Ladislav Bocak

The list of supplementary materials.

Table S1. The list of the sequences used in the mtDNA and rRNA phylogenetic analysis (Fig. 1A).
Table S2. Primers used for PCR amplifications and PCR settings.
Table S3. Distances among sampled localities in the Main Range of the Malay Peninsula (km).
Table S4. Number of identified mtDNA haplotypes and intraspecific uncorrected paired genetic distances for the Malay clade.
Table S5. Uncorrected ("p") distance matrix for haplotypes of the *rrnL* mtDNA fragment.
Table S6. Uncorrected ("p") distance matrix for haplotypes of the *cox1-3'* mtDNA fragment.
Table S7. Uncorrected ("p") distance matrix for haplotypes of the nad5 mtDNA fragment.
Table S8. Per-individual read numbers (Reads), number of clusters (Clusters), and mean coverage depth (Mean depth) for the NextRAD data.
Table S9. Percentage of SNP bases identical for *S. pseudoumbrosus*, *S. pahangensis*, and *S. anthracinus.*

Figure S1. Sampling sites in the Main Range in Peninsular Malaysia.
Figure S2. Climate of the Main Range in Peninsular Malaysia.
Figure S3. Density plot for nuclear inter- and intraspecific pairwise similarity.
Figure S4. How variation in minimum coverage depth affects heterozygosity and proportion of loci accepted into the dataset.
Figure S5. Run information for testing of the effect of clustering threshold on the within-individual heterozygosity and the number of the loci generated.
Figure S6. Principal Component Analysis of two nuclear datasets.
Figure S7. Phylogenetic hypothesis derived from the three-species nuclear dataset (the putative hybrid included/excluded).
Figure S8. Timing of the speciation events in the Malay clade.
Figure S9. Diversity of the male genitalia in the genus *Scarelus.*

Table S1. The list of the sequences used in the mtDNA and rRNA phylognetic analysis (Fig. 1A).

Species	Geographic origin	Fragments rrnL	18S	28S	cox1-3'	nad5	Voucher Number*
Ourgroup (the Sumatran and Javan clades, as in Malohlava & Bocak 2010)							
S. brastagiensis	Sumatra, Utara, Brastagi, Gn. Sibayak	HM451006	HM451131	HM451088	HM451047	HM451217	VM0012
	Sumatra, Utara, Brastagi, Gn. Sibayak	HM451007	HM451132	HM451089	HM451048	HM451218	VM0013
S. flavicolis	Sumatra, Jambi, Kersik Tua. Gn. Kerinci	HM451020	HM451148	HM451103	HM451060	HM451231	VM0030
	Sumatra, Jambi, Kersik Tua. Gn. Kerinci	HM451021	HM451149	HM451104	HM451061	HM451232	VM0031
	Sumatra, Jambi, Kersik Tua. Gn. Kerinci	HM451022	HM451150	HM451105	HM451062	HM451233	VM0032
	Sumatra, Jambi, Kersik Tua. Gn. Kerinci	HM451025	HM451155	HM451110	-	HM451238	VM0037
S. longicornis	Sumatra, Gn. Talamau	HM451010	HM451135	HM451091	-	HM451220	VM0017
	Sumatra, Barat Lake, Maninjau	HM451011	HM451136	HM451092	-	HM451221	VM0018
	Sumatra, Barat Lake, Maninjau	HM451017	HM451143	HM451098	-	HM451226	VM0025
	Sumatra, Barat Lake, Maninjau	HM451018	HM451144	HM451099	HM451057	HM451227	VM0026
	Sumatra, Barat Lake, Maninjau	HM451019	HM451145	HM451100	-	HM451228	VM0027
S. rufus	Sumatra, Jambi, Kersik Tua, Gn. Kerinci	HM451024	HM451152	HM451107	HM451064	HM451235	VM0034
S. ruficollis	Sumatra, Jambi Prov, Kerinci Seblat N.P.	-	HM451127	HM451085	HM451078	HM451213	VM0008
	Sumatra, Jambi, Kersik Tua, Gn. Kerinci		HM451147	HM451102	HM451059	HM451230	VM0029
	Sumatra, Jambi, Kersik Tua, Gn. Kerinci	HM451023	HM451151	HM451106	HM451063	HM451234	VM0033
	Sumatra, Jambi, Kersik Tua, Gn. Kerinci	-	HM451153	HM451108	HM451065	HM451236	VM0035
	Sumatra, Jambi, Kersik Tua, Gn. Kerinci	-	HM451154	HM451109	HM451066	HM451237	VM0036
S. sanguineus	Sumatra, Gn. Merapi, 5 km of Kotobaru	-	HM451122	HM451080	HM451039	HM451208	VM0003
	Sumatra, Gn. Merapi, 5 km of Kotobaru	-	HM451128	HM451086	HM451044	HM451214	VM0009
	Sumatra, Barat, 10 km of Bukittinggi	HM451016	HM451141	HM451096	HM451055	HM451224	VM0023
	Sumatra, Sumatra, Barat, Bukittinggi	-	HM451142	HM451097	HM451056	HM451225	VM0024
S. saranganensis	Java, C, Gn. Lawu, 8 km W of Sarangan	HM451005	HM451130	HM451087	HM451046	HM451216	VM0011
	Java, C, Gn. Lawu, 8 km W of Sarangan	HM451004	HM451129	-	HM451045	HM451215	VM0010
S. cibodasensis	Java, W, Puncak Pass	HM451003	HM451126	HM451084	HM451043	HM451212	VM0007
Ingroup (the Malay clade)							
S. umbrosus	Malaysia, Perak, Rd.Tapah-Ringlet km24	KU319589	-	-	KU319617	KU319644	NG0060

	Malaysia, Perak, Rd.Tapah-Ringlet km34	KU319590	-	-	KU319618	KU319645	NG0058
	Malaysia, Perak, Rd.Tapah-Ringlet km40	KU319591	-	-	KU319619	KU319646	NG0057
S. anthracinus	Malaysia, Pahang, Cameron Highl.	HM450998	HM451120	---	HM451037	HM451206	VM0001
	Malaysia, Pahang, Cameron Highl.	HM451002	HM451125	HM451083	HM451042	HM451211	VM0006
	Malaysia, Pahang, Cameron Highl.	HM451009	HM451134	HM451090-	HM451050	HM451219	VM0016
	Malaysia, Pahang, Cameron Highl.	HM451026	HM451156	HM451111	HM451067	HM451239	VM0038
	Malaysia, Pahang, Tanah Rata, Gn. Jasar	HM451027	HM451157	---	HM451068	HM451240	VM0039
	Malaysia, Pahang, Tanah Rata, Gn. Jasar	HM451028	HM451158	HM451112	HM451069	HM451241	VM0040
	Malaysia, Pahang, Tanah Rata, Gn. Jasar	HM451029	HM451159	HM451113	HM451070	HM451242	VM0043
	Malaysia, Pahang, Tanah Rata, Gn. Jasar	HM451030	HM451160	HM451114	HM451071	HM451243	VM0044
	Malaysia, Pahang, Tanah Rata, Gn. Jasar	HM451031	HM451161	HM451115	HM451072	HM451244	VM0045
	Malaysia, Pahang, Tanah Rata, Gn. Jasar	HM451032	HM451162	HM451116	HM451073	HM451245	VM0046
	Malaysia, Pahang, Tanah Rata, Gn. Jasar	HM451033	HM451163	HM451117	HM451074	HM451246	VM0047
	Malaysia, Pahang, Tanah Rata, Gn. Jasar	HM451034	HM451164		HM451075	HM451247	VM0048
	Malaysia, Pahang, Gn. Jasar, E slope	KU319592	-	-	KU319629	-	NG0025
	Malaysia, Pahang, Gn. Jasar, E slope	KU319593	-	-	KU319623	KU319650	NG0026
	Malaysia, Pahang, Gn. Jasar, E slope	KU319598	-	-	KU319640	-	NG0042
	Malaysia, Pahang, Gn. Jasar, N slope	KU319594	-	-	KU319633	-	NG0054
	Malaysia, Pahang, Gn.Beremban N slope	KU319596	-	-	-	KU319651	NG0027
	Malaysia, Pahang, Gn.Beremban N slope	KU319597	-	-	-	KU319652	NG0028
	Malaysia, Pahang, Gn.Beremban N slope	-	-	-	-	KU319653	NG0029
	Malaysia, Pahang, Gn.Beremban N slope	-	-	-	-	KU319654	NG0030
	Malaysia, Pahang, Gn.Beremban N slope	KU319600	-	-	KU319624	-	NG0043
	Malaysia, Pahang, Gn.Beremban N slope	KU319601	-	-	KU319625	KU319655	NG0044
	Malaysia, Pahang, Gn.Beremban N slope	KU319602	-	-	KU319627	KU319656	NG0045
	Malaysia, Pahang, Gn.Beremban N slope	KU319603	-	-	KU319626	KU319657	NG0046
	Malaysia, Pahang, Gn.Beremban	-	-	-	-	KU319658	NG0031
	Malaysia, Pahang, Gn.Beremban	KU319605	-	-	-	KU319659	NG0032
	Malaysia, Pahang, Gn.Beremban	-	-	-	-	KU319660	NG0033
	Malaysia, Pahang, Gn.Beremban	KU319607	-	-	KU319630	KU319661	NG0041
	Malaysia, Pahang, Gn.Beremban	KU319608	-	-	KU319631	-	NG0047
	Malaysia, Pahang, Gn.Beremban	KU319609	-	-	KU319632	KU319662	NG0048
	Malaysia, Pahang, Gn.Beremban	KU319595	-	-	KU319634	KU319663	NG0049
	Malaysia, Pahang, Gn.Brinchang	-	-	-	-	KU319664	NG0034
	Malaysia, Pahang, Gn.Brinchang	-	-	-	KU319635	KU319665	NG0035
	Malaysia, Pahang, Gn.Brinchang	KU319599	-	-	KU319636	KU319671	NG0036
	Malaysia, Pahang, Gn.Brinchang	KU319610	-	-	KU319637	KU319666	NG0037
	Malaysia, Pahang, Gn.Brinchang	KU319611	-	-	KU319628	KU319667	NG0050
	Malaysia, Pahang, Gn.Brinchang	KU319613	-	-	KU319622	KU319668	NG0051
	Malaysia, Pahang, Gn.Brinchang	KU319612	-	-	KU319638	KU319669	NG0052
	Malaysia, Pahang, Gn.Brinchang	KU319606	-	-	KU319639	KU319670	NG0053

S. pahangensis	Malaysia, Pahang, Kampong Kuala Boh	-	HM451123	HM451081	HM451040	HM451209	VM0004
	Malaysia, Pahang, Kampong Kuala Boh	KU319614	-	-	KU319621	-	NG0055
	Malaysia, Pahang, Kampong Kuala Boh	KU319620	-	-	KU319620	KU319649	NG0056
S. pseudoumbrosus	Malaysia, W Pahang, Road Ipoh, Kg. Raja	HM450999	HM451121	HM451079	HM451038	HM451207	VM0002
	Malaysia, Pahang, Gn. Jasar N slope	KU319604	-	-	KU319641	KU319647	NG0038
	Malaysia, Perak, Rd.Ipoh-Kg.Raja km47	KU319615	-	-	KU319642	-	NG0059
	Malaysia, Perak, Rd.Ipoh-Kg.Raja km47	KU319616	-	-	KU319643	KU319648	NG0062

*The full GenBank Voucher Numbers consist of 'UPOL'+ a code given here

Table S2. Primers used for PCR amplifications and PCR settings

Fragment	Code	-mer	Sequence (5' >> 3')
18S rRNA	5'	24	GACAACCTGGTTGATCCTGCCAGT
	b5.0	19	TAACCGCAACAACTTTAAT
	ai	22	CCTGAGAAACGGCTACCACATC
	b2.5	20	TCTTTGGCAAATGCTTTCGC
	a1.0	20	GGTGAAATTCTTGGACCGTC
	bi	20	GAGTCTCGTTCGTTATCGGA
	3'I	24	CACCTACGGAAACCTTGTTACGAC
	a2.0	19	ATGGTTGCAAAGCTGAAAC
28S rRNA	ff	20	TTACACACTCCTTAGCGGAT
	dd	19	GGGACCCGTCTTGAAACAC
rrnL mtDNA	16a	20	CGCCTGTTTAACAAAAACAT
	16b	22	CCGGTCTGAACTCAGATCATGT
	ND1A	27	GGTCCCTTACGAATTTGAATATATCCT
cox1 mtDNA	JerM	23	CAACAYYTATTTTGRTTYTTTGG
	Pat	25	TCCATTGCACTAATCTGCCATATTA
	Marilyn	21	TCATAAGTTCAGTATCATTG
	Marcy	27	TARTTCRTATGWTCAATAYCAYTGRTG
nad5 mtDNA	OF1	29	CCTACTCCTGTTTCTGCTTTAGTTCATTC
	R6	29	GAAACGAAAAATCGTATTTAATTTCGACT

PCR was performed using 0.5-0.6 U Taq polymerase, 1 mM $MgCl_2$, 50 mM each dNTP, 0.2 mM primer, and typically 0.03 mg of template in 50 ml reaction volume. Cycle conditions were generally 2 min at 94°C, 30-60 sec at 94°C, 30-60 sec at 45-52°C (depending on the melting temperatures of primer pairs used), 1-2 min at 72°C (repeated for 30-40 cycles), and 10 min at 72°C. ABI technology was used for DNA sequencing and sequences were edited using Sequencher 4.0.5 software (Gene Codes Corp.).

Table S3. Distances among sampled localities in the Main Range of the Malay Peninsula (km)

		1	2	3	4	5	6	7	8	9	10
1	Gunung Brinchang	-									
2	G. Jasar, E slope	4.41	-								
3	G. Jasar, N slope	3.79	1.08	-							
4	G. Beremban, N slope	2.56	2.89	2.90	-						
5	G. Beremban	5.05	2.67	3.37	2.50	-					
6	Kampong Kuala Boh	12.14	11.78	12.47	10.21	9.13	-				
7	Rd. Ipoh-Kg. Raja km 47	12.19	14.48	13.48	14.49	16.50	24.18	-			
8	Rd. Tapah-Ringlet km 40	13.15	9.08	10.08	10.73	8.28	11.88	23.15	-		
9	Rd. Tapah-Ringlet km 34	16.38	12.00	12.82	14.34	12.12	16.77	24.59	4.86	-	
10	Rd. Tapah-Ringlet km 24	21.68	17.31	18.08	19.53	17.20	20.51	29.63	9.24	5.27	-

Table S4. Number of identified mtDNA haplotypes and intraspecific uncorrected paired genetic distances for the Malay clade

rrnL fragment.	*S. umbrosus*	3 haplotypes, intraspecific variability <0.76%,
	S. pseudoumbrosus	3 haplotypes, intraspecific variability <0.76%
	S. pahangensis	1 haplotype
	S. anthracinus	11 unique haplotypes, intraspecific variability <0.38%
cox1-3' fragment	S. *umbrosus*	3 haplotypes, intraspecific variability <1.90%,
	S. pseudoumbrosus	3 haplotypes, intraspecific variability <3.17%
	S. pahangensis	2 haplotype, intraspecific variability <0.45%
	S. anthracinus	17 haplotypes, intraspecific variability <1.63%
nad5 + tRNAs fragment	*S. umbrosus*	3 haplotypes, intraspecific variability <2.19%,
	S. pseudoumbrosus	4 haplotypes, intraspecific variability <2.61%
	S. pahangensis	1 haplotype
	S. anthracinus	17 unique haplotypes, intraspecific variability <1.09%

Table S5. Uncorrected ("p") distance matrix for haplotypes of the *rrnL* fragment

	NG0058	NG0057	NG0060	VM0001	NG0041	NG0044	NG0053	NG0056	VM0002	NG0062	NG0059
NG0058 *S. umbrosus*	-										
NG0057 *S. umbrosus*	0.00761	-									
NG0060 *S. umbrosus*	0.00761	0.00508	-								
VM0001 *S. anthracinus*	0.11862	0.11606	0.11478	-							
NG0041 *S. anthracinus*	0.11568	0.11303	0.11296	0.00134	-						
NG0044 *S. anthracinus*	0.11729	0.11474	0.11347	0.00256	0.00133	-					
NG0053 *S. anthracinus*	0.11984	0.11729	0.11601	0.00127	0.00266	0.00383	-				
NG0056 *S. pahangensis*	0.12114	0.12114	0.11987	0.02552	0.02392	0.02296	0.02679	-			
VM0002 *S. pseudoumbrosus*	0.10461	0.10715	0.10587	0.03185	0.03060	0.03190	0.03317	0.03828	-		
NG0062 *S. pseudoumbrosus*	0.10835	0.11089	0.10962	0.03185	0.02928	0.03063	0.03190	0.03698	0.00764	-	
NG0059 *S. pseudoumbrosus*	0.10837	0.11092	0.10965	0.03062	0.02926	0.03061	0.03189	0.03699	0.00638	0.00000	-

Table S6. Uncorrected ("p") distance matrix for haplotypes of the *cox1-3'* mtDNA fragment

	VM0016	VM0044	VM0043	VM0001	NG0025	NG0045	NG0051	NG0052	NG0035	NG0037	NG0055	VM0004	VM0059	VM0038
VM0016 *S. anthracinus*	-													
VM0044 *S. anthracinus*	0.00547	-												
VM0043 *S. anthracinus*	0.00365	0.00182	-											
VM0001 *S. anthracinus*	0.00365	0.00912	0.00729	-										
NG0025 *S. anthracinus*	0.00365	0.00912	0.00729	0.00000	-									
NG0045 *S. anthracinus*	0.01185	0.01003	0.00820	0.01550	0.01550	-								
NG0051 *S. anthracinus*	0.01094	0.00912	0.00729	0.01459	0.01459	0.00091	-							
NG0052 *S. anthracinus*	0.01185	0.01003	0.00820	0.01550	0.01550	0.00182	0.00091	-						
NG0035 *S. anthracinus*	0.01276	0.00729	0.00912	0.01641	0.01641	0.01003	0.00912	0.01003	-					
NG0037 *S. anthracinus*	0.01280	0.00732	0.00915	0.01646	0.01646	0.01005	0.00914	0.01005	0.00000	-				
NG0055 *S. pahangensis*	0.06108	0.05743	0.05925	0.06199	0.06199	0.06016	0.05925	0.06016	0.05925	0.05860	-			
VM0004 *S. pahangensis*	0.06433	0.06062	0.06245	0.06526	0.06526	0.06332	0.06238	0.06330	0.06249	0.06256	0.00468			
NG0059 *S. pseudoumbrosus*	0.10027	0.09754	0.09754	0.10027	0.10027	0.10027	0.09936	0.10027	0.10119	0.10154	0.10027	0.10346	-	
NG0038 *S. pseudoumbrosus*	0.09889	0.09615	0.09615	0.09889	0.09889	0.09888	0.09797	0.09888	0.09980	0.09982	0.09982	0.10464	0.00183	-
VM0002 *S. pseudoumbrosus*	0.09201	0.08926	0.08926	0.09293	0.09293	0.09385	0.09293	0.09385	0.09294	0.09298	0.08925	0.09357	0.03127	0.02948
NG0057 *S. umbrosus*	0.18152	0.18060	0.18060	0.18151	0.18151	0.18152	0.18061	0.18152	0.17878	0.17960	0.18422	0.18317	0.17783	0.17778
NG0058 *S. umbrosus*	0.17954	0.17862	0.17862	0.17953	0.17953	0.17953	0.17862	0.17954	0.17680	0.17760	0.18678	0.18497	0.17767	0.17761
NG0060 *S. umbrosus*	0.18317	0.18225	0.18225	0.18316	0.18316	0.18317	0.18226	0.18317	0.18044	0.18125	0.18677	0.18495	0.17858	0.17852

Uncorrected ("p") distance matrix (continued)

	VM0002	NG0057	NG0058	NG0060
VM0002 *S. pseudoumbrosus*	-			
NG0057 *S. umbrosus*	0.17583	-		
NG0058 *S. umbrosus*	0.17567	0.01545	-	
NG0060 *S. umbrosus*	0.17841	0.01636	0.01907	-

Table S7. Uncorrected ("p") distance matrix for haplotypes of the *nad5* mtDNA fragment

	NG0043	NG0048	NG0046	VM0039	VM0001	NG0025	NG0053	VM0038	NG0042	VM0045	NG0026	VM0016	NG0035	NG0037
NG0043 *S. anthracinus*	-													
NG0048 *S. anthracinus*	0.00000	-												
NG0046 *S. anthracinus*	0.00000	0.00000	-											
VM0039 *S. anthracinus*	0.00169	0.00264	0.00169	-										
VM0001 *S. anthracinus*	0.00254	0.00388	0.00254	0.00254	-									
NG0025 *S. anthracinus*	0.00339	0.00515	0.00339	0.00339	0.00085	-								
NG0053 *S. anthracinus*	0.00339	0.00512	0.00339	0.00339	0.00085	0.00169	-							
VM0038 *S. pseudoumbrosus*	0.00593	0.00788	0.00593	0.00424	0.00339	0.00424	0.00424	-						
NG0042 *S. anthracinus*	0.00508	0.00661	0.00508	0.00508	0.00254	0.00339	0.00339	0.00085	-					
VM0045 *S. anthracinus*	0.00593	0.00660	0.00593	0.00593	0.00339	0.00424	0.00424	0.00169	0.00085	-				
NG0026 *S. anthracinus*	0.00593	0.00662	0.00593	0.00593	0.00339	0.00424	0.00424	0.00169	0.00085	0.00169	-			
VM0016 *S. anthracinus*	0.00678	0.00925	0.00678	0.00593	0.00424	0.00508	0.00508	0.00169	0.00169	0.00254	0.00254	-		
NG0035 *S. anthracinus*	0.00848	0.01025	0.00848	0.00848	0.00594	0.00678	0.00593	0.00933	0.00849	0.00764	0.00933	0.01018	-	
NG0037 *S. anthracinus*	0.00848	0.01025	0.00848	0.00848	0.00594	0.00678	0.00593	0.00933	0.00849	0.00764	0.00933	0.01018	0.00000	-
NG0050 *S. anthracinus*	0.00848	0.01026	0.00848	0.00848	0.00594	0.00679	0.00594	0.00934	0.00850	0.00765	0.00934	0.01020	0.00000	0.00000
VM0046 *S. anthracinus*	0.00848	0.01025	0.00848	0.00848	0.00594	0.00678	0.00593	0.00933	0.00849	0.00764	0.00933	0.01018	0.00000	0.00000
VM0044 *S. anthracinus*	0.00933	0.01151	0.00933	0.00763	0.00678	0.00763	0.00678	0.00849	0.00933	0.00849	0.01018	0.01018	0.00085	0.00085
VM0043 *S. anthracinus*	0.00848	0.01035	0.00848	0.00678	0.00593	0.00678	0.00593	0.00764	0.00848	0.00764	0.00933	0.00933	0.00509	0.00509
NG0052 *S. anthracinus*	0.00932	0.01043	0.00932	0.00932	0.00678	0.00763	0.00678	0.01018	0.00933	0.00848	0.01018	0.01103	0.00594	0.00594
NG0045 *S. anthracinus*	0.00848	0.01043	0.00848	0.00848	0.00593	0.00678	0.00593	0.00933	0.00848	0.00764	0.00933	0.01018	0.00509	0.00509
NG0051 *S. anthracinus*	0.00932	0.01043	0.00932	0.00932	0.00678	0.00763	0.00678	0.01018	0.00933	0.00848	0.01018	0.01103	0.00594	0.00594
NG0055 *S. pahangensis*	0.04495	0.04286	0.04495	0.04665	0.04665	0.04750	0.04665	0.04835	0.04750	0.04665	0.04835	0.04920	0.04580	0.04580
VM0004 *S. pahangensis*	0.04495	0.04286	0.04495	0.04665	0.04665	0.04750	0.04665	0.04835	0.04750	0.04665	0.04835	0.04920	0.04580	0.04580
VM0002 *S. pseudoumbrosus*	0.06612	0.06621	0.06612	0.06527	0.06442	0.06527	0.06442	0.06443	0.06443	0.06358	0.06528	0.06443	0.06443	0.06443
NG0059 *S. pseudoumbrosus*	0.07882	0.07203	0.07882	0.08052	0.07883	0.07968	0.07882	0.08052	0.07968	0.07883	0.08053	0.08137	0.07882	0.07882
NG0038 *S. pseudoumbrosus*	0.08052	0.07461	0.08052	0.08221	0.08052	0.08137	0.08052	0.08137	0.08053	0.07968	0.08138	0.08222	0.08052	0.08052
NG0062 *S. pseudoumbrosus*	0.07967	0.07335	0.07967	0.08137	0.07967	0.08052	0.07967	0.08052	0.07968	0.07883	0.08053	0.08137	0.07967	0.07967
NG0058 *S. umbrosus*	0.21855	0.21234	0.21855	0.21854	0.21686	0.21771	0.21686	0.21854	0.21770	0.21855	0.21770	0.21939	0.21874	0.21874
NG0060 *S. umbrosus*	0.21517	0.20962	0.21517	0.21516	0.21348	0.21432	0.21348	0.21432	0.21348	0.21432	0.21348	0.21516	0.21536	0.21536
NG0057 *S. umbrosus*	0.21449	0.21077	0.21449	0.21448	0.21278	0.21363	0.21278	0.21447	0.21363	0.21448	0.21362	0.21531	0.21468	0.21468

Uncorrected ("p") distance matrix (continued)

	NG0050	VM0046	VM0044	VM0043	NG0052	NG0045	NG0051	NG0055	VM0004	VM0002	NG0059	NG0038	NG0062	NG0058	NG0060	NG0057
NG0050 *S. anthracinus*	-															
VM0046 *S. anthracinus*	0.00000	-														
VM0044 *S. anthracinus*	0.00085	0.00085	-													
VM0043 *S. anthracinus*	0.00509	0.00509	0.00424	-												
NG0052 *S. anthracinus*	0.00594	0.00594	0.00679	0.00254	-											
NG0045 *S. anthracinus*	0.00510	0.00509	0.00594	0.00170	0.00085	-										
NG0051 *S. anthracinus*	0.00595	0.00594	0.00679	0.00254	0.00170	0.00085	-									
NG0055 *S. pahangensis*	0.04584	0.04580	0.04665	0.04580	0.04665	0.04580	0.04495	-								
VM0004 *S. pahangensis*	0.04584	0.04580	0.04665	0.04580	0.04665	0.04580	0.04495	0.00000	-							
VM0002 *S. pseudoumbrosus*	0.06450	0.06443	0.06443	0.06358	0.06358	0.06273	0.06189	0.07460	0.07460	-						
NG0059 *S. pseudoumbrosus*	0.07889	0.07882	0.07967	0.07882	0.07798	0.07713	0.07628	0.08222	0.08222	0.02623	-					
NG0038 *S. pseudoumbrosus*	0.08059	0.08052	0.08137	0.08052	0.07967	0.07882	0.07798	0.08307	0.08307	0.02623	0.00169	-				
NG0062 *S. pseudoumbrosus*	0.07974	0.07967	0.08052	0.07967	0.07883	0.07798	0.07713	0.08222	0.08222	0.02538	0.00085	0.00085	-			
NG0058 *S. umbrosus*	0.21893	0.21874	0.21958	0.21618	0.21619	0.21534	0.21534	0.22221	0.22221	0.22331	0.22670	0.22839	0.22755	-		
NG0060 *S. umbrosus*	0.21555	0.21536	0.21620	0.21280	0.21281	0.21196	0.21196	0.22138	0.22138	0.21909	0.22586	0.22586	0.22501	0.02194	-	
NG0057 *S. umbrosus*	0.21487	0.21468	0.21552	0.21212	0.21213	0.21128	0.21128	0.21793	0.21793	0.21925	0.22264	0.22434	0.22349	0.01772	0.01604	-

Supplementary Table S8. Per-individual read numbers (Reads), number of clusters (Clusters), and mean coverage depth (Mean depth) for the NextRAD data.

Individual	Reads	Clusters	Mean depth
NG0025	2677088	113477	14.132
NG0026	2215212	110911	11.801
NG0035	2871738	117564	14.787
NG0036	3804707	107688	20.488
NG0037	2917374	82728	20.602
NG0038	2439179	68353	21.591
NG0041	2309301	73883	18.104
NG0042	2428190	70138	19.653
NG0043	2578357	105748	14.555
NG0044	1944212	80870	13.569
NG0045	2103973	103848	11.838
NG0046	2166833	95386	12.669
NG0047	3259136	87222	22.166
NG0048	2389299	69847	19.805
NG0049	2978779	68167	24.971
NG0050	2447961	75515	18.081
NG0051	2680044	96863	16.586
NG0052	2723272	92650	17.837
NG0053	2677789	93932	17.189
NG0054	3229917	104959	17.765
NG0055	2495466	108952	14.230
NG0056	2251016	87744	15.516
NG0057	2871731	112177	14.475
NG0058	1893228	107695	9.2330
NG0059	2456478	113341	13.315
NG0060	3734553	91057	21.718
NG0061	2110629	110012	11.505
NG0062	2291879	110464	12.487

Table S8. Percentage of SNP bases identical for *S. pseudoumbrosus, S. pahangensis, and S. anthracinus* (25 sample dataset). Individual NG0038 is a putative hybrid (see the main taxt for further information)

		NG0062	NG0059	NG0055	NG0056	NG0038	NG0049	NG0061	NG0041	NG0047	NG0048	NG0025	NG0026	NG0042	NG0054	NG0043	NG0044	NG0035	NG0050	NG0036	NG0046	NG0037	NG0045	NG0053	NG0052	NG0051
Scarelus pseudoumbrosus	NG0062		75.13%	74.41%	73.26%	75.87%	77.36%	75.92%	77.05%	78.13%	76.80%	77.25%	75.80%	76.37%	77.56%	77.57%	75.81%	77.23%	77.33%	77.51%	75.88%	78.48%	75.93%	77.49%	77.00%	76.50%
	NG0059	75.13%		74.10%	72.92%	75.37%	76.89%	75.50%	76.81%	77.91%	76.22%	76.97%	75.26%	75.98%	77.00%	77.20%	74.97%	76.96%	76.72%	77.59%	75.47%	78.22%	75.21%	77.15%	76.85%	76.34%
Scarelus pahangensis	NG0055	74.41%	74.10%		75.92%	75.72%	77.75%	76.88%	77.94%	79.08%	77.46%	77.73%	76.22%	76.68%	77.80%	77.98%	75.94%	77.87%	77.44%	78.15%	76.19%	79.35%	76.27%	77.90%	77.76%	76.95%
	NG0056	73.26%	72.92%	75.92%		74.90%	76.59%	75.64%	76.68%	78.03%	76.50%	76.36%	75.22%	75.54%	76.83%	76.93%	75.04%	76.79%	76.45%	77.28%	75.41%	78.15%	75.34%	76.58%	76.41%	76.14%
	NG0038	75.87%	75.37%	75.72%	74.90%		81.46%	79.69%	81.24%	82.91%	81.37%	82.19%	80.39%	80.84%	82.19%	82.19%	80.69%	82.53%	82.04%	82.49%	80.58%	83.55%	80.35%	82.08%	81.85%	81.46%
Scarelus anthracinus	NG0049	77.36%	76.89%	77.75%	76.59%	81.46%		82.88%	84.56%	86.13%	84.22%	84.90%	82.96%	84.12%	84.88%	85.23%	83.16%	85.06%	84.66%	85.25%	83.49%	86.21%	83.25%	84.96%	85.05%	84.00%
	NG0061	75.92%	75.50%	76.88%	75.64%	79.69%	82.88%		82.78%	84.07%	82.79%	82.90%	81.15%	82.07%	83.28%	83.24%	81.58%	83.18%	82.83%	83.55%	81.62%	84.61%	81.56%	83.25%	82.92%	82.40%
	NG0041	77.05%	76.81%	77.94%	76.68%	81.24%	84.56%	82.78%		86.21%	84.82%	85.14%	83.36%	83.93%	85.22%	85.17%	83.27%	85.51%	84.70%	85.77%	83.88%	86.53%	83.57%	85.31%	85.10%	84.24%
	NG0047	78.13%	77.91%	79.08%	78.03%	82.91%	86.13%	84.07%	86.21%		86.15%	86.41%	85.17%	85.42%	86.84%	86.89%	84.45%	86.75%	86.20%	87.29%	85.27%	88.43%	84.90%	86.63%	86.54%	85.64%
	NG0048	76.80%	76.22%	77.46%	76.50%	81.37%	84.22%	82.79%	84.82%	86.15%		84.82%	83.82%	83.91%	84.95%	85.56%	83.41%	85.21%	84.78%	85.55%	83.61%	86.47%	83.66%	85.21%	85.44%	84.33%
	NG0025	77.25%	76.97%	77.73%	76.36%	82.19%	84.90%	82.90%	85.14%	86.41%	84.82%		83.56%	84.29%	85.53%	85.47%	83.62%	85.71%	85.32%	86.33%	84.09%	87.10%	83.67%	85.88%	85.74%	84.60%
	NG0026	75.80%	75.26%	76.22%	75.22%	80.39%	82.96%	81.15%	83.36%	85.17%	83.82%	83.56%		83.16%	84.12%	84.12%	82.41%	84.16%	83.53%	84.67%	82.40%	85.58%	82.45%	83.98%	84.20%	82.97%
	NG0042	76.37%	75.98%	76.68%	75.54%	80.84%	84.12%	82.07%	83.93%	85.42%	83.91%	84.29%	83.16%		84.40%	85.06%	83.03%	84.45%	84.14%	84.85%	83.30%	85.64%	83.03%	84.74%	84.63%	83.68%
	NG0054	77.56%	77.00%	77.80%	76.83%	82.19%	84.88%	83.28%	85.22%	86.84%	84.95%	85.53%	84.12%	84.40%		86.13%	83.89%	86.11%	85.60%	86.23%	84.01%	87.47%	83.77%	85.83%	85.61%	84.85%
	NG0043	77.57%	77.20%	77.98%	76.93%	82.19%	85.23%	83.24%	85.17%	86.89%	85.56%	85.47%	84.12%	85.06%	86.13%		84.38%	86.30%	85.59%	86.33%	84.52%	87.17%	84.24%	86.05%	86.00%	85.18%
	NG0044	75.81%	74.97%	75.94%	75.04%	80.69%	83.16%	81.58%	83.27%	84.45%	83.41%	83.62%	82.41%	83.03%	83.89%	84.38%		84.06%	83.78%	84.61%	82.66%	85.37%	82.41%	83.87%	84.03%	83.09%
	NG0035	77.23%	76.96%	77.87%	76.79%	82.53%	85.06%	83.18%	85.51%	86.75%	85.21%	85.71%	84.16%	84.45%	86.11%	86.30%	84.06%		85.68%	86.69%	84.55%	87.75%	84.36%	86.00%	86.07%	85.15%
	NG0050	77.33%	76.72%	77.44%	76.45%	82.04%	84.66%	82.83%	84.70%	86.20%	84.78%	85.32%	83.53%	84.14%	85.60%	85.59%	83.78%	85.68%		85.62%	83.99%	86.95%	84.00%	85.66%	85.29%	85.05%
	NG0036	77.51%	77.59%	78.15%	77.28%	82.49%	85.25%	83.55%	85.77%	87.29%	85.55%	86.33%	84.67%	84.85%	86.23%	86.33%	84.61%	86.69%	85.62%		84.88%	87.62%	84.53%	86.32%	86.24%	85.38%
	NG0046	75.88%	75.47%	76.19%	75.41%	80.58%	83.49%	81.62%	83.88%	85.27%	83.61%	84.09%	82.40%	83.30%	84.01%	84.52%	82.66%	84.55%	83.99%	84.88%		85.56%	82.71%	84.40%	84.33%	83.87%
	NG0037	78.48%	78.22%	79.35%	78.15%	83.55%	86.21%	84.61%	86.53%	88.43%	86.47%	87.10%	85.58%	85.64%	87.47%	87.17%	85.37%	87.75%	86.95%	87.62%	85.56%		85.45%	87.59%	87.34%	86.64%
	NG0045	75.93%	75.21%	76.27%	75.34%	80.35%	83.25%	81.56%	83.57%	84.90%	83.66%	83.67%	82.45%	83.03%	83.77%	84.24%	82.41%	84.36%	84.00%	84.53%	82.71%	85.45%		84.31%	83.85%	83.29%
	NG0053	77.49%	77.15%	77.90%	76.58%	82.08%	84.96%	83.25%	85.31%	86.63%	85.21%	85.88%	83.98%	84.74%	85.83%	86.05%	83.87%	86.00%	85.66%	86.32%	84.40%	87.59%	84.31%		85.82%	84.96%
	NG0052	77.00%	76.85%	77.76%	76.41%	81.85%	85.05%	82.92%	85.10%	86.54%	85.44%	85.74%	84.20%	84.63%	85.61%	86.00%	84.03%	86.07%	85.29%	86.24%	84.33%	87.34%	83.85%	85.82%		84.80%
	NG0051	76.50%	76.34%	76.95%	76.14%	81.46%	84.00%	82.40%	84.24%	85.64%	84.33%	84.60%	82.97%	83.68%	84.85%	85.18%	83.09%	85.15%	85.05%	85.38%	83.87%	86.64%	83.29%	84.96%	84.80%	

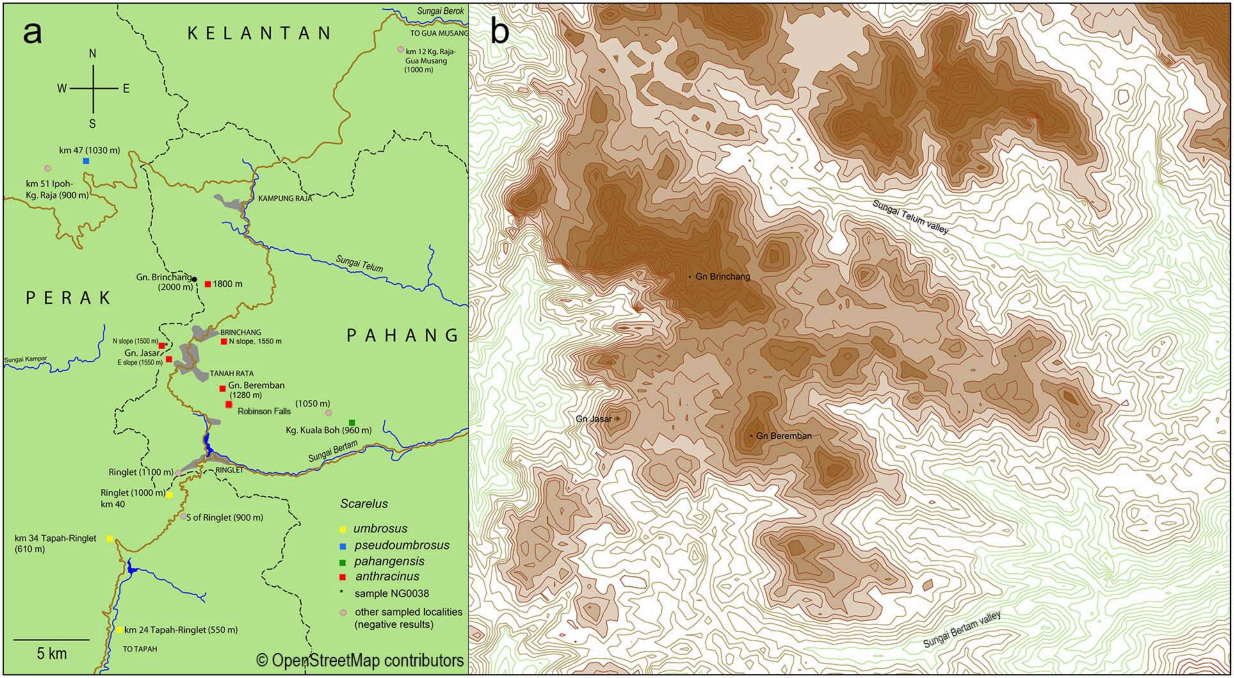

Figure S1. (a) Sampling sites in the Main Range in the Malay Peninsula. The map was derived from OpenStreetMap® open data, licensed under the Open Data Commons Open Database License (ODbL) by the OpenStreetMap Foundation (OSMF).

The cartography in the OpenStreetMap map tiles is licensed under CC BY-SA (www.openstreetmap.org/copyright). The licence terms can be found on the following link: http://creativecommons.org/licenses/by-sa/2.0/

(b) Topographical map of the study area. The map was produced using the QGIS software package (www.qgis.org/en/site/forusers/download.html) from ASTER GDEM V2 data. ASTER GDEM V2 data are available free of charge to users worldwide from the Land Processes Distributed Active Archive Center (LP DAAC) and J-spacesystems (https://asterweb.jpl.nasa.gov/gdem.asp). The hypsometric scale was produced with the Photoshop CS6 software package.

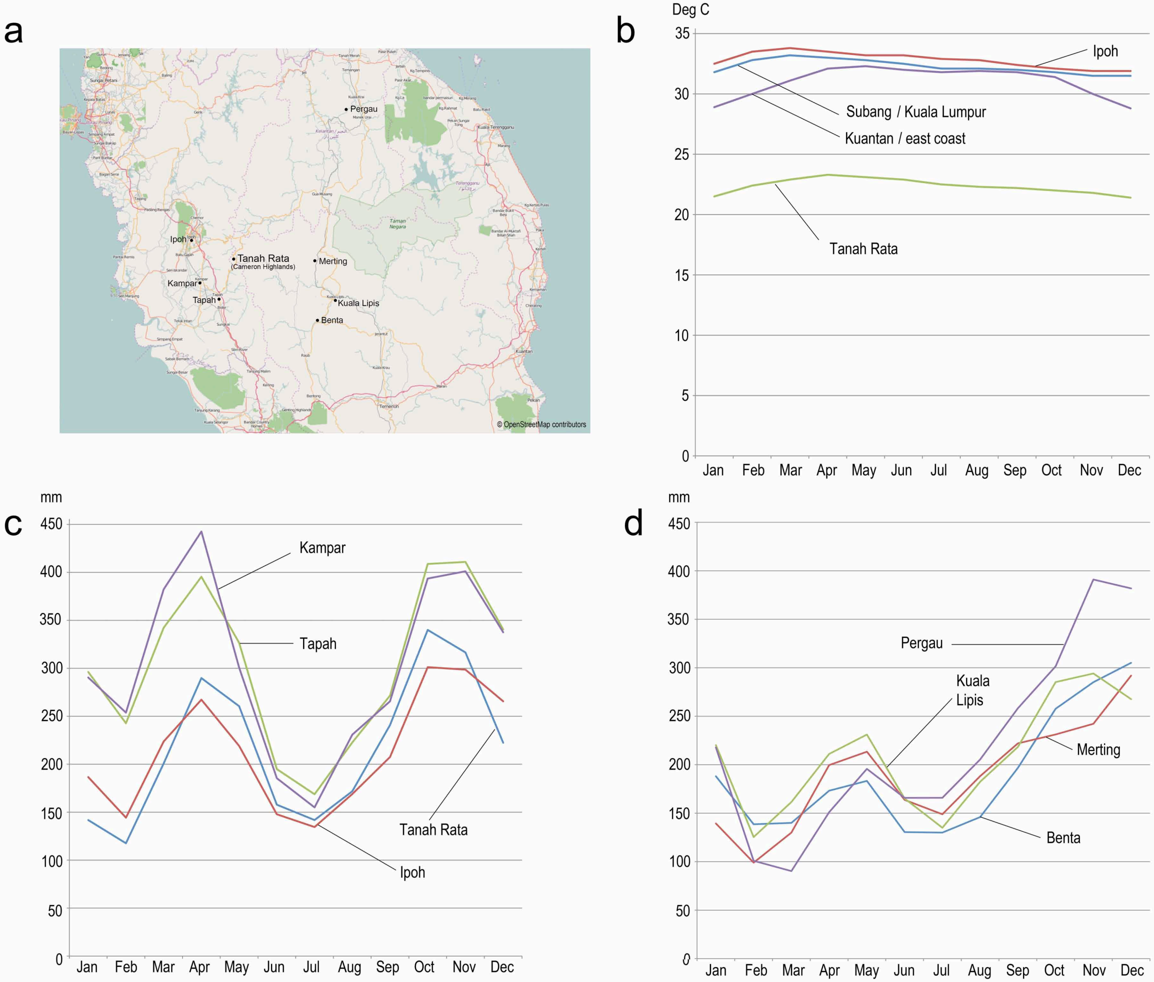

Figure S2. (a) Positions of meterological stations. The map was derived from OpenStreetMap® open data, licensed under the Open Data Commons Open Database License (ODbL) by the OpenStreetMap Foundation (OSMF). The cartography in the OpenStreetMap map tiles is licensed under CC BY-SA (www.openstreetmap.org/copyright). The licence terms can be found on the following link: http://creativecommons.org/licenses/by-sa/2.0/
The map was uploaded from the openstreetmap.org server and positions of meterological station were marked in the Photoshop CS6 software.

(b) Average maximum temperature.

(c) Monthly average rainfall in the Cameron Highlands, and the western slope of the Main Range.

(d) Monthly avwrage rainfall on the eastern slope of the Main Range.

Data taken from the Global historical Climatology network Database (www.worldclimate.com).

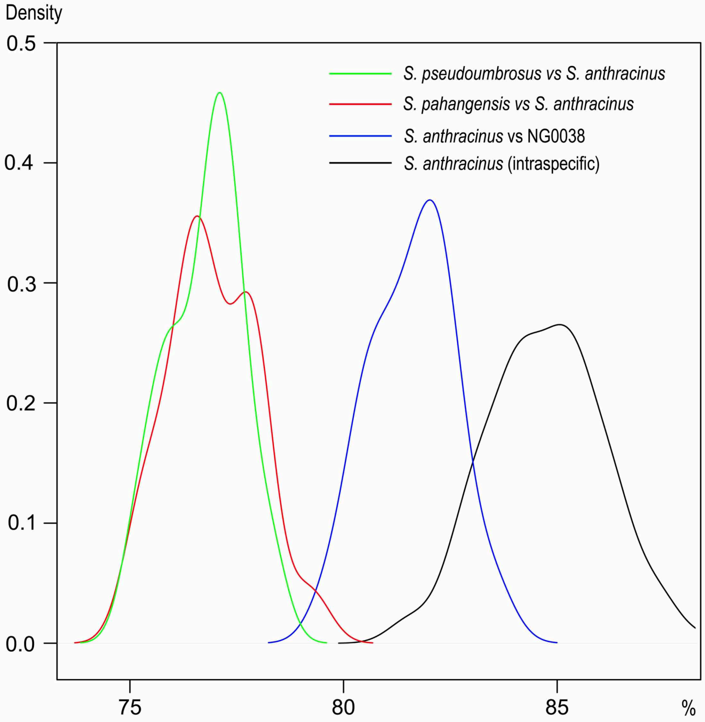

Figure S3. Density plot for nuclear inter- and intraspecific pairwise similarity inferred from the three-species dataset. The specimen NG0038 is a putative hybrid (see the main text for further information).

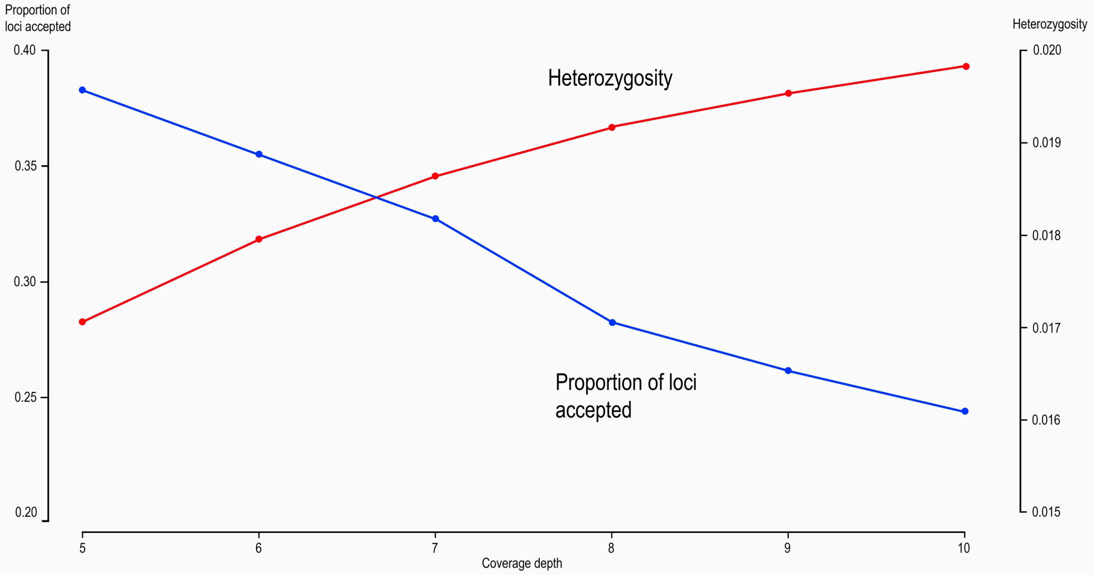

Figure S4. How variation in minimum coverage depth affects heterozygosity and the proportion of loci accepted into the dataset

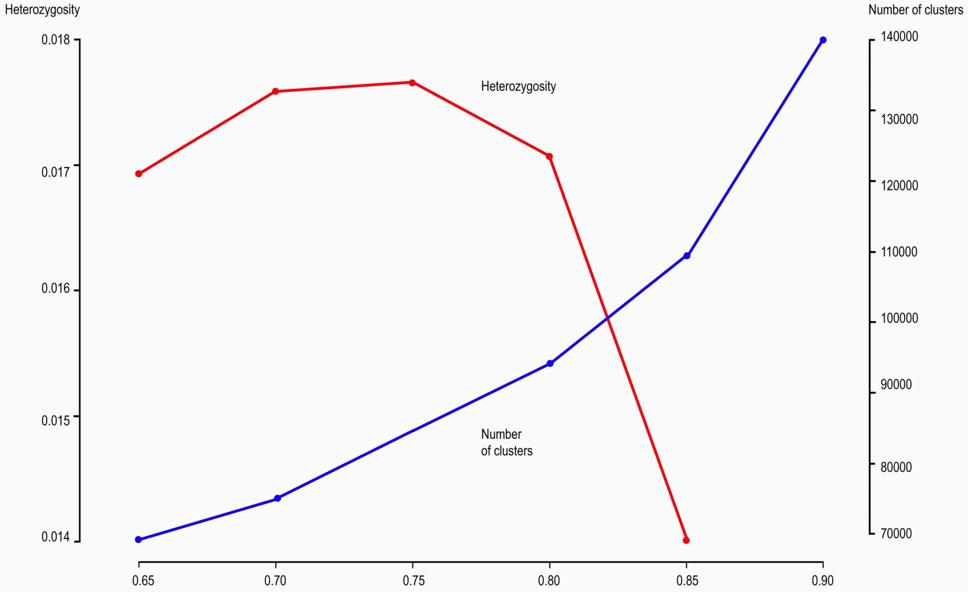

Figure S5. Run information for testing of the effect of clustering threshod on the within-individual heterozygosity and the number ofthe loci generated.

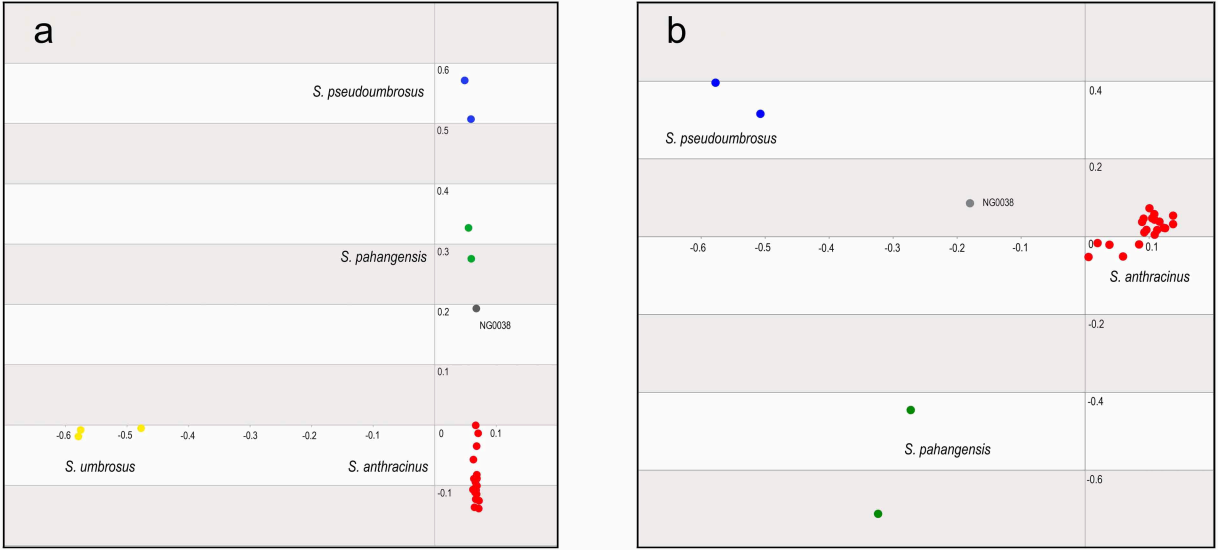

Figure S6. The Principal Component Analyses of two nuclear datasets. (a) complete dataset with *S. umbrosus* included. (b) dataset without *S. umbrosus.* Principal Component 1 (x-axis) explains 15% and 9 %, and Component 2 explains 7% and 7% of the total variation for A and B, respectively. The sample NG0038 is a non-identified putative hybrid.

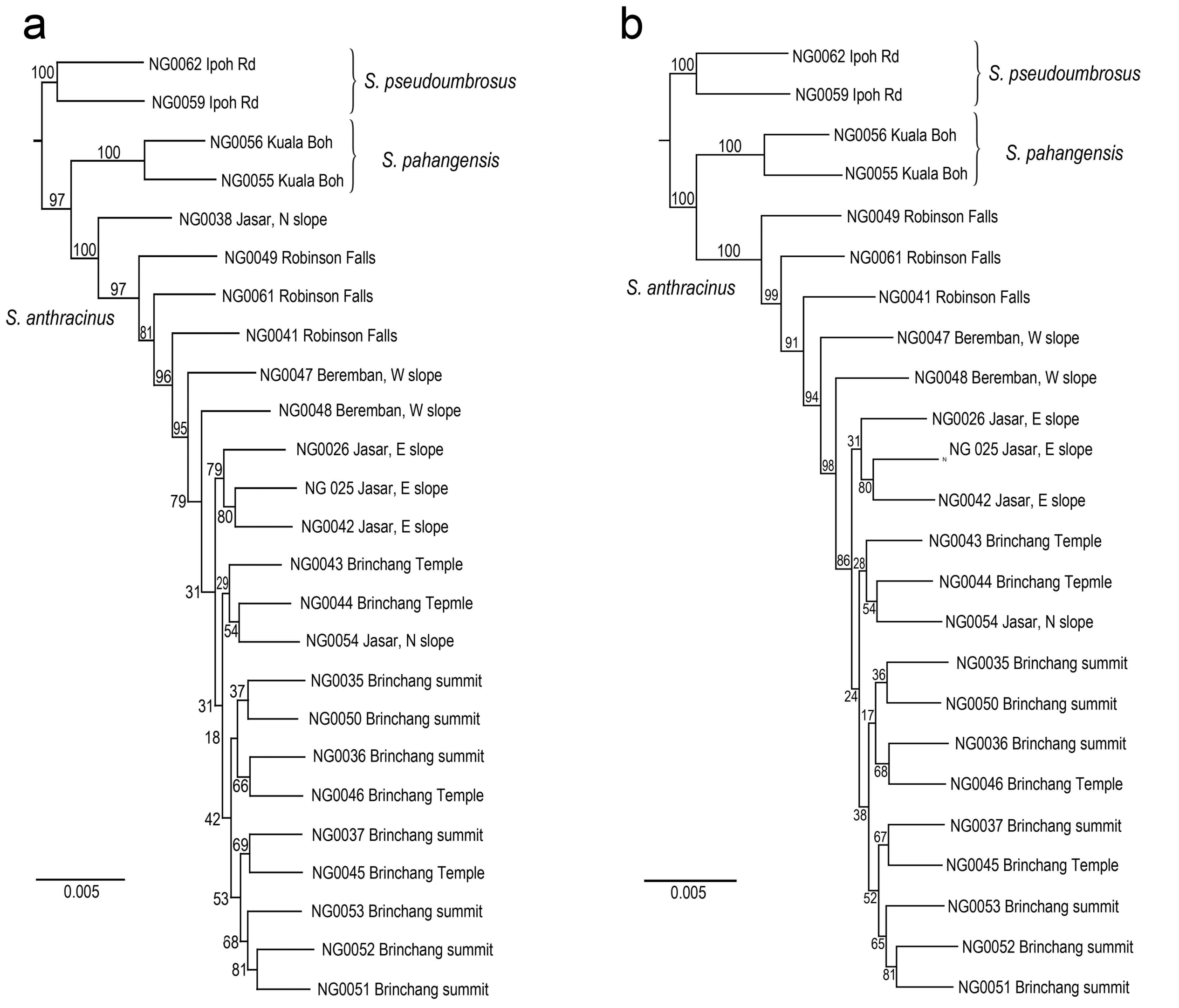

Figure S7. The phylogenetic hypothesis derived by the maximum likelihood analysis from the three species dataset of the RAD loci. (a) 25-sample dataset; (b) 24-sample dataset with the putative hybrid (sample NG0038) excluded. The values at branches designate bootstrap supports. The Figure 7b as Fig. 2d.

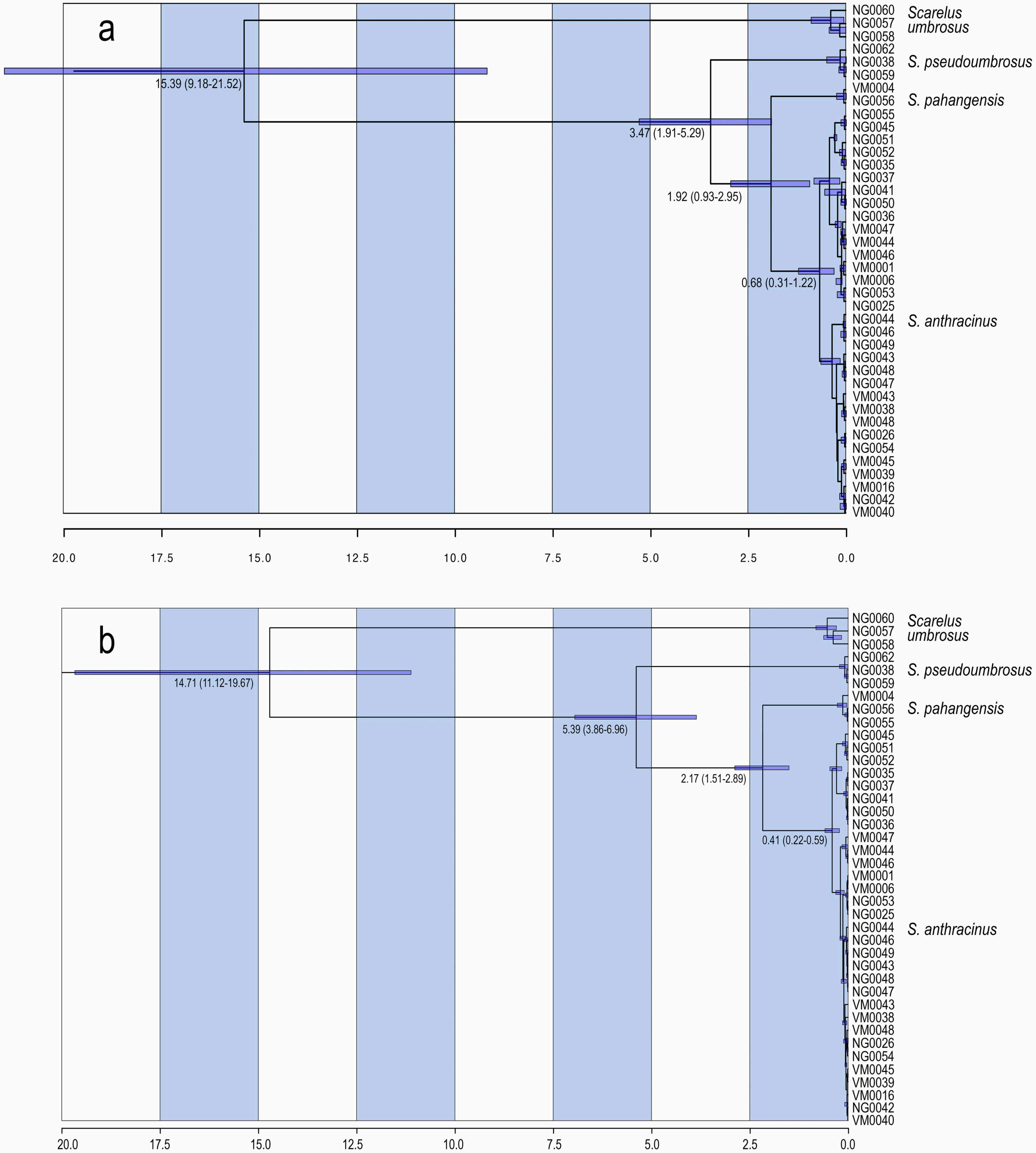

Figure S8. Timing of the speciation events in the clade of the Malay *Scarelus* species inferred using the Beast package. (a) the *rrnL* mt DNA dataset, (b) the *cox1* mtDNA dataset.

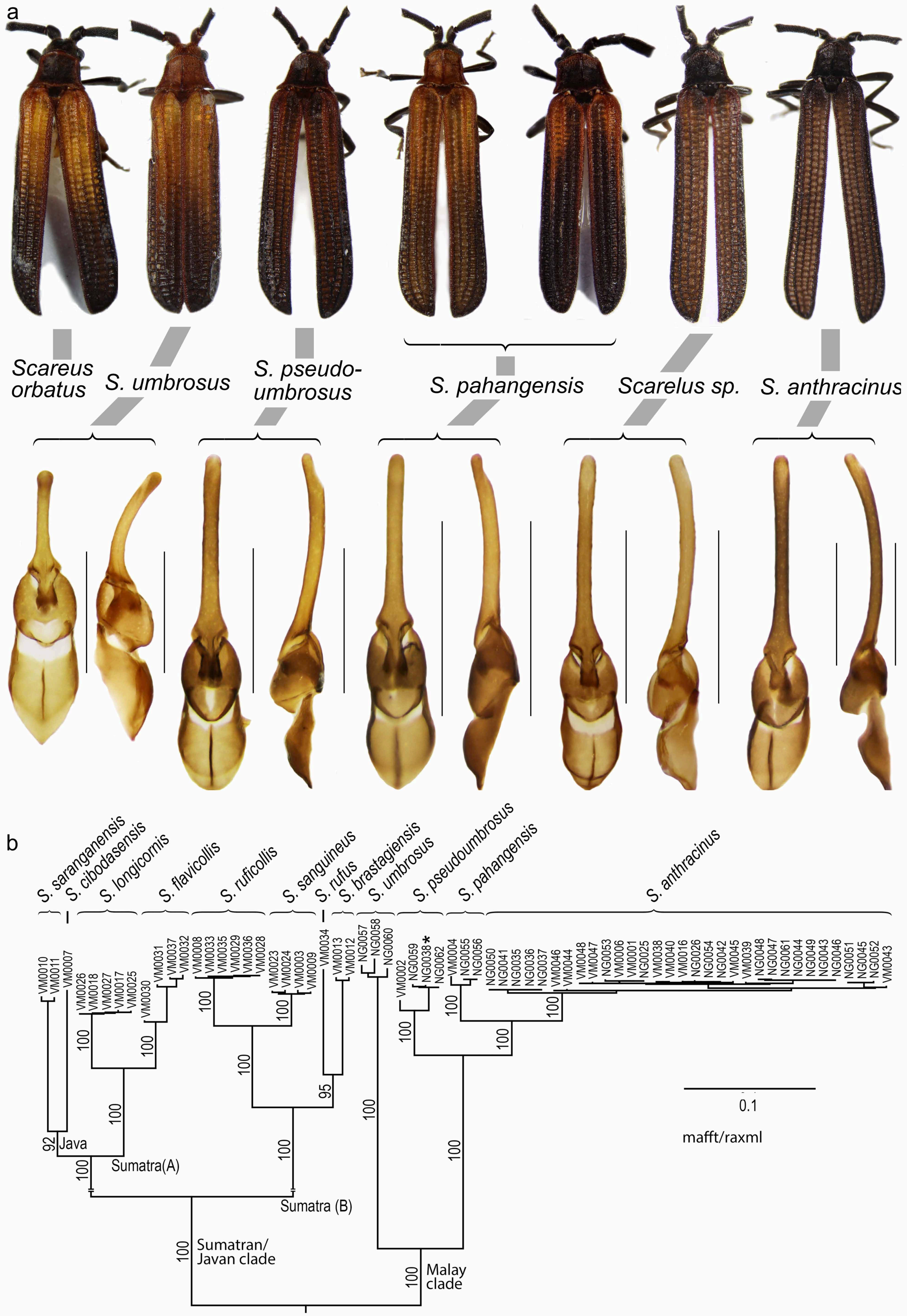

Figure S9. (a) General appearance and male genitalia of Malay *Scarelus*; *S. orbatus* is a species closely related to *S. longicornis* in Fig. S9b; (b) Phylogenetic hypothesis for *Scarelus* based on a maximum likelihood analysis of five fragments obtained by Sanger sequencing and aligned using MAFFT algorithm; the Bornean and Philippine clades omitted, the numbers at branches indicate bootstrap support.

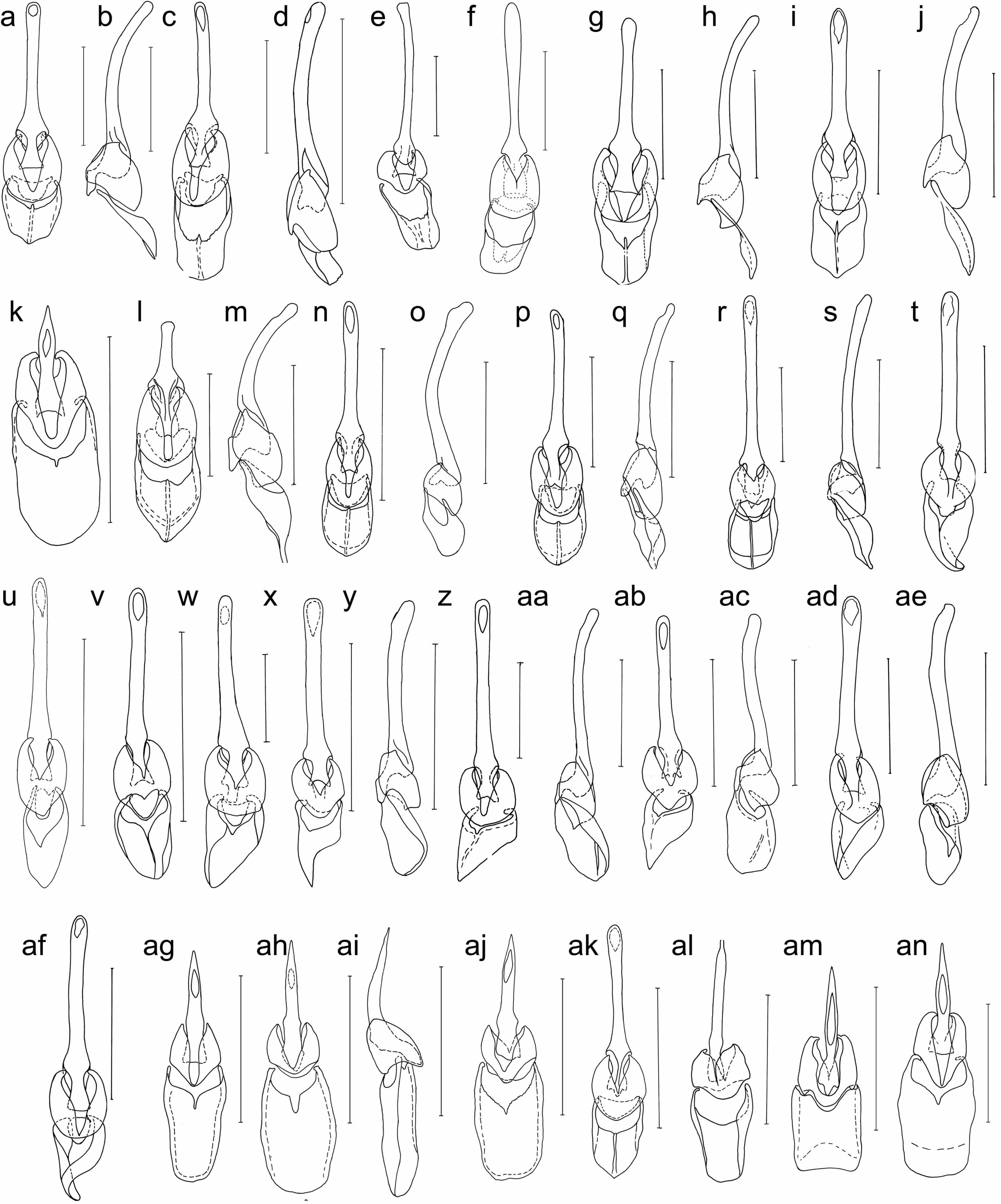

Figure S10. Male genitalia. (a-b) *S. ruficollis*, (c-d) *S. brastagiensis*, (e) *S. tomani*, (f) *S. schawalleri*, (g-h) *S. sanguineus*, (i-j) *S. rufus*, (k) *S. orbatus*, (l-m) *S. umbrosus*, (m) *S. pahangensis*, (n) *S. ardens*, (p-q), *S. pseudoumbrosus*, (r-s) *S. anthracinus*, (t) *S. baranciki*, (u) *S. kodadai*, (v) *S. palawensis*, (w) *S. crudus*, (x-y) *S. bicostatus*, (z-aa) *S. rollei*, (ab-ac) *S. similis*, (ad) *S. loksadoensis* , (ae-af) *S. salvani*, (ag) *S. sarangensis*, (ah-ai) *S. cibodasensis*, (aj) *S. javanus*, (ak) *S. inapicalis*, (al) *S. corporaali*, (am) *S. longicornis*, (an) *S. flavicollis*. Scales 0.5 mm.
